# Pulmonary Group 2 Innate Lymphoid Cell Phenotype Is Context Specific: Determining the Effect of Strain, Location, and Stimuli

**DOI:** 10.3389/fimmu.2019.03114

**Published:** 2020-01-22

**Authors:** Lewis J. Entwistle, Lisa G. Gregory, Robert A. Oliver, William J. Branchett, Franz Puttur, Clare M. Lloyd

**Affiliations:** Inflammation, Repair and Development, National Heart and Lung Institute, Imperial College London, London, United Kingdom

**Keywords:** group 2 innate lymphoid cell, allergen, airway, lung, phenotype, marker expression, BALB/c, C57BL/6

## Abstract

Group 2 innate lymphoid cells (ILC2s) are enriched at mucosal sites, including the lung, and play a central role in type 2 immunity and maintaining tissue homeostasis. As a result, since their discovery in 2010, research into ILC2s has increased markedly. Numerous strategies have been used to define ILC2s by flow cytometry, often utilizing different combinations of surface markers despite their expression being variable and context-dependent. In this study, we sought to generate a comprehensive characterization of pulmonary ILC2s, identifying stable and context specific markers from different pulmonary compartments following different airway exposures in different strains of mice. Our analysis revealed that pulmonary ILC2 surface marker phenotype is heterogeneous and is influenced by mouse strain, pulmonary location, *in vivo* treatment/exposure and *ex vivo* stimulation. Therefore, we propose that a lineage negative cell expressing CD45 and Gata3 defines an ILC2, and subsequent surface marker expression should be used to describe their phenotype in context-specific scenarios.

## Introduction

In 2010, three papers were published simultaneously describing an innate cell type lacking known lineage markers which produce the archetypal type 2 cytokine IL-13 (1–3). Subsequently innate lymphoid cells (ILCs) were broadly classified into 3 groups reflecting the innate counterpart of adaptive T cells (4).

ILCs develop from common innate lymphoid progenitors which first start to populate tissues during fetal development. Group 2 ILCs (ILC2s), first described as “nuocytes” and “innate type 2 cells,” are considered the innate counterpart of Th2 cells, requiring Gata3 expression for production of the hallmark type 2 cytokines; IL-5 and IL-13. ILC2s are predominantly found at mucosal sites and are responsive to epithelial alarmin cytokines (5, 6), such as IL-33, IL-25, and TSLP, producing IL-5 and IL-13 (2, 7) and inducing different transcriptional profiles (8). ILC2s have also been demonstrated to produce IL-4, but this requires leukotriene D_4_ (9, 10). Once activated, ILC2s require IL-9 for survival in the lung (11). Depending on the tissue, resident ILC2s are responsive to and activated by different alarmins; gut-resident ILC2s respond to IL-25, lung and fat resident ILC2s respond to IL-33 and skin resident ILC2s respond to TSLP (12). Furthermore, ILC2s are closely associated with nerves in mucosal tissues, with neuron-derived neuropeptides demonstrated to augment ILC2 activation. The neuropeptide Neuromedin U (NMU) can act on ILC2s via the NMU receptor 1 (NMUR1) to induce type 2 cytokine production *in vitro* as well as amplify type 2 inflammation *in vivo* (8, 13, 14). Conversely, the neuropeptide calcitonin gene-related peptide (CGRP) negatively regulates ILC2 activation and effector functions triggered by alarmins and NMU (15, 16). Although ILC2s were originally thought to be seeded in and restricted to specific tissues, recent evidence has prompted discussion as to whether ILC2s are truly pre-adapted to a specific tissue (17) or migrate from other sites and adapt to the new tissue (18, 19). For example, at steady state natural ILC2s (nILC2s), which express ST2 and are therefore IL-33 responsive, are present in the lungs of mice but, under inflammatory conditions inflammatory ILC2s (iILC2s), which lack ST2 expression but express IL-17RB and are therefore responsive to IL-25 (18), can be recruited from the intestinal lamina propria to the lung and contribute to host defense. These data not only suggests the existence of two ILC2 subpopulations, but also demonstrates that ILC2s can orchestrate local and distant tissue protection (20). ILCs also exhibit plasticity and ILC2s have been shown both *in vitro* and *in vivo* to convert to ILC1-like cells expressing T-bet and the IL-12Rc (21–24).

Regardless of their migratory and adaptive capacity, the ability of ILC2s to produce vast quantities of type 2 cytokines places them at the center of type 2 immune responses. Indeed, ILC2s have been shown to be required and sufficient for the expulsion of intestinal helminths (5, 10) and driving allergic lung inflammation (25–27), as well as maintaining tissue homeostasis (28). ILC2s have been demonstrated to facilitate Th2 differentiation, activation, and expansion through the production of IL-4 and IL-2 (10, 29), PD-L1:PD-1 interactions (30) and OX40L expression (31). T cells are also important for ILC2 maintenance following activation, through IL-2 production (32).

With their central role in type 2 immunity, research into ILC2s has led to numerous published strategies to define an ILC2 (Table 1), often using combinations of extracellular markers and intracellular cytokine or transcription factor expression (40). However, expression of cell surface markers by ILC2s is variable and context-dependent (37) and transcription factor or cytokine expression often requires a reporter mouse. The purpose of this study was to identify stable and context specific markers of ILC2s from different pulmonary compartments following different airway exposures in different strains of mice. Here we used rIL-33, commonly used to induce ILC2s in the lungs *in vivo*, and two different clinically relevant allergens, house-dust mite (HDM) and *Alternaria alternata* (ALT), in two strains of mice frequently used for laboratory studies, C57BL/6 and BALB/c mice.

**Table 1:** Published gating strategies to define pulmonary ILC2s.

**ILC2 definition**	**Mouse background**	**Model**	**Year**	**References**
^†^Lin^−^ cKit^+^ Sca-1^+^	C57BL/6	Helminth infection (Gut)	2010	(1)
^†^Lin^−^ ST2^+^ IL-17BR^+^	BALB/c^*^	rIL-33, rIL-25, Helminth infection (Gut)	2010	(2)
Lin^−^ CD25^+^ CD127^+^ ST2^+^ Sca-1^+^	C57BL/6,^*^ BALB/c	rIL-33	2013	(33)
Lin^−^ ST2^+^ KLRG1^+^	C57BL/6	rIL-33, rIL-25	2015	(18)
Lin^−^ CD127^+^ Sca-1^+^ ST2^+^ KLRG1^+^	C57BL/6	n/a	2015	(34)
Lin^−^ ICOS^+^ IL-13^+^	BALB/c^*^	rIl-33, HDM,	2015	(27)
Lin^−^ CD25^+^ ST2^+^	C57BL/6	rIL33, rIL-2 + rIL-25	2016	(35)
Lin^−^ cKit^+^ CD90.2^+^	C57BL/6^*^	rIL-33	2016	(35)
Lin^−^ MHCII^−^ CD25^+^ ST2^+^	C57BL/6^*^	Helminth infection	2016	(35)
Lin^−^ KLRG1^+^ CD90.2^+^ ST2^+^ CD25^+^	C57BL/6^*^	Helminth infection	2016	(35)
Lin^−^ CD90.2^+^ KLRG1^+^ ST2^+^	C57BL/6^*^	Helminth infection	2016	(35)
Lin^−^ KLRG1^+^ Sca-1^+^	C57BL/6	Helminth infection	2016	(35)
Lin^−^ KLRG1^+^ ICOS^+^	C57BL/6^*^	rIL-25, rIL-25 + IFN-γ	2016	(35)
Lin^−^ CD127^+^ Gata3^+^	C57BL/6	n/a	2017	(36)
Lin^−^*Gata3*^YFP+^	C57BL/6^*^	rIL-33, HDM	2017	(37)
Lin^−^ CD127^+^ Gata3^+^	C57BL/6^*^	rIL-33, rIL-25, TSLP, papain, LPS	2018	(31)
Lin^−^ Sca-1^+^ Gata3^+^	C57BL/6	Influenza x31 H3N2, HDM or both	2018	(38)
Lin^−^ CD90.2^+^ IL-13^+^	BALB/c	rIL-33, HDM, ALT	2018	(39)

† *Original description of ILC2s*.

* *Transgenic mice used*.

## Materials and Methods

### Animals and Reagents

WT female BALB/c and C57BL/6 were obtained from Charles River (Saffron Walden, UK). *Il13*^eGFP^ mice, that contain GFP tagged IL-13 protein, were generously gifted by Andrew McKenzie. Mice were maintained in specific pathogen–free conditions and given food and water *ad libitum*. In all experiments, mice were maintained under the same environmental conditions and were randomly assigned treatment groups. All procedures were conducted in accordance with the Animals Scientific Procedures Act (ASPA) 1986. Recombinant mouse IL-33 for intranasal administration was purchased from Biolegend UK.

### Intranasal Challenge

Mice received either house dust mite (HDM) (25 μg), *Alternaria alternata* (ALT) (10 μg), rIL-33 (50 μg/kg), or phosphate buffered saline (PBS) three times a week for 2 weeks, all in 25 μL. Animals were culled 24 h following the final allergen challenge.

### Cell Recovery

Airway lumen cells were recovered by Bronchoalveolar lavage (BAL). The lumen was flushed with 3 × 400 μl PBS via a tracheal cannula. Following lavage, the mediastinal lymph node (mLN) was located and removed into complete RPMI media [10% fetal calf serum, 2 mM l-glutamine, and penicillin/streptomycin (100 U/ml)]. Cells were then retrieved by filtering through a 70 μm sieve (Falcon, BD Biosciences, MA). All the lung lobes were removed, finely chopped and incubated at 37°C in complete RPMI containing collagenase (0.15 mg/ml; Type D, Roche Diagnostics) and deoxyribonuclease (25 mg/ml; Type 1, Roche Diagnostics) for 1 h. Cells were recovered by the same method as mLN. The red blood cells were then lysed and the remaining cells washed and re-suspended ready for analysis.

### Flow Cytometry

To stain for intracellular cytokines, ~4.5 × 10^6^ total cells were either incubated with PMA (20 ng/ml; Sigma-Aldrich), ionomycin-free acid (1.5 μg/ml) from Streptomyces conglobatus (Merck), and Brefeldin A (5 μg/ml; Sigma-Aldrich) or with Brefeldin A (5 μg/ml) alone or complete media (200 μL) at 37°C for 4 h. Cells were then washed in PBS and stained with LIVE/DEAD Fixable Blue Dead Cell Stain (Thermo Fisher Scientific/Life Technologies) for 20 min at 4°C. To stain for extracellular antigens, cells were incubated for 20 min at 4°C with anti-mouse CD16/32 (FC) block (BD Biosciences) and antibodies (Table S1) in PBS containing 1% bovine serum albumin and 0.01% sodium azide. Cells were then re-suspended in Foxp3 fixation/permeabilization buffer (Thermo Fisher Scientific/eBioscience) according to the manufactures instructions and left overnight at 4°C (Up to 18 h). For experiments using *Il13*^eGFP^ mice, cells fixed using 1% paraformaldehyde. The following morning cells were re-suspended in permeabilization buffer and left at room temperature for 5 min. Intracellular markers were then stained in permeabilization buffer for 30 min at 4°C. Cells were washed twice and re-suspended in PBS ready for analysis. Data were acquired with a BD LSR Fortessa with FACSDIVA software (BD Biosciences) and analyzed using FlowJo software (v10, Tree Star). To assist with analysis, fluorescence minus one (FMO) controls for antigens were used. The lineage exclusion cocktail consisted of CD11b, CD11c, CD19, CD3e, CD5, F4/80, FCεR1, GR-1, NKP46, TCR-β, TCRγδ, and TER-119. See Table S1 for all details of all anti-mouse antibodies used.

### Statistical Analysis

All results were expressed as mean ± SEM, and data were analyzed using GraphPad Prism 7 software (GraphPad Software). Sidak's and Tukey's two-way ANOVA tests were used to detect differences between groups, or Mann Whitney when appropriate. Statistical significance was accepted when *P* < 0.05. ^*/†^*P* < 0.05, ^**/††^*P* < 0.01, ^***^/^†††^*P* < 0.001, and ^****^/^††††^*P* < 0.0001.

## Results

### Identification of Pulmonary ILC2s Induced Following Allergen or rIL-33 Treatment

**Figure 1:**
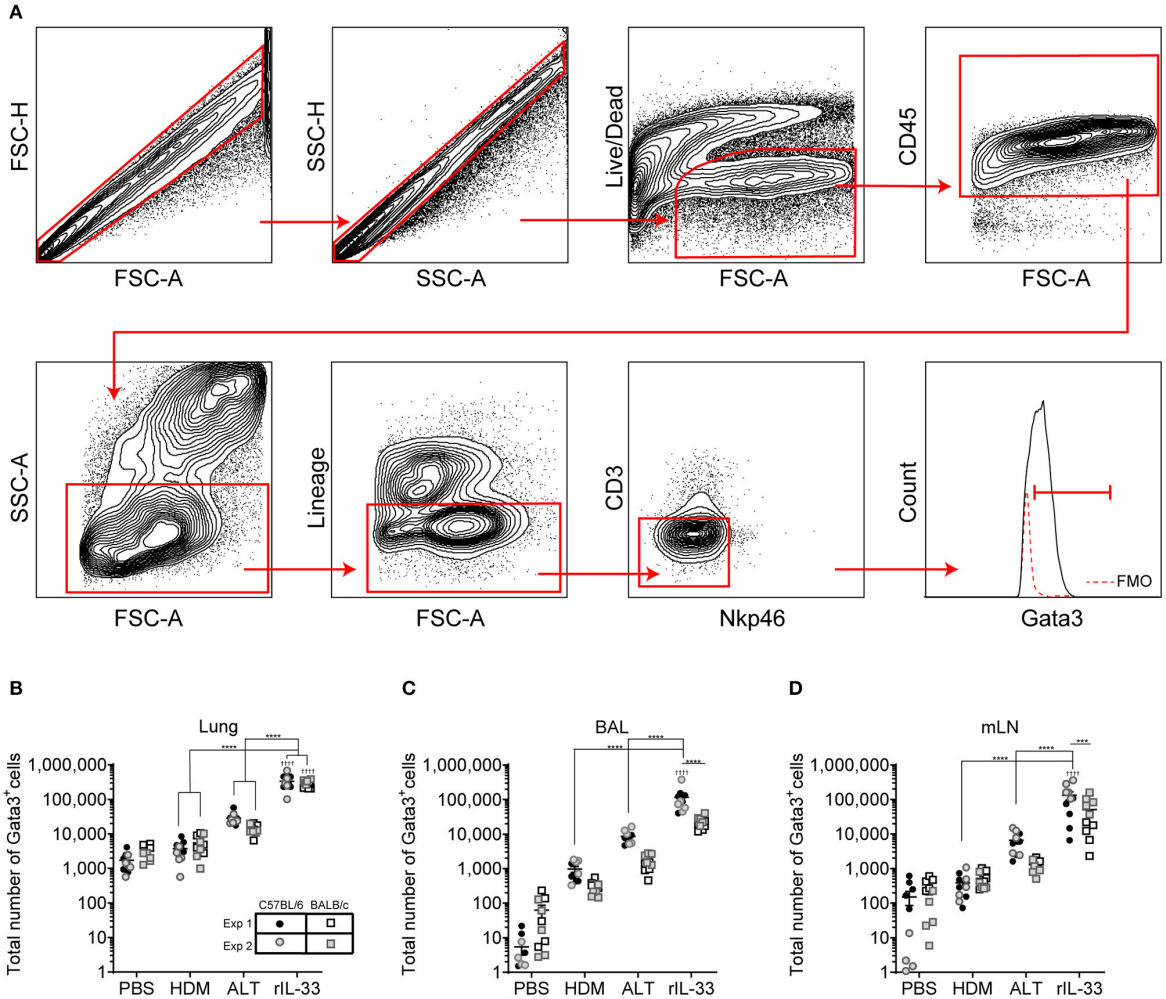
Group 2 innate cells increase in response to allergen and rIL-33. **(A)** Gating Schematic. ILC2s are defined as Single, Live, CD45^+^, Lymphoid, Lineage^neg^ (TCRβ, TCRγδ, CD3e, CD5, CD19, CD11b, CD11c, FCεR1, GR-1, F4/80, and TER-119 negative), CD3^−^, Nkp46^−^, Gata3^+^. **(B)** Total number of Gata3^+^ cells in the Lineage^neg^ CD3^−^, NKp46^−^ population in the lung. **(C)** BAL and **(D)** Mediastinal lymph node (mLn). Data is the combination of two individual experiments, *n* = 10 per group. ^††††^*P* < 0.0001 compared to PBS control. ***P < 0.001, ****P < 0.0001 comparing between strains or treatment groups.

Following airway exposure to HDM, ALT, rIL-33, or PBS control, ILC2s were identified by flow cytometry; single, live cells, expressing CD45 within the lymphocyte gate and not expressing lineage (Lin) markers (CD5, CD11b, CD11c, CD19, TCRβ, TCRγδ, GR-1, F4/80, FCεR1, TER-119), CD3 or Nkp46 were then gated on for positive expression of the transcription factor Gata3 (Figure 1A). It was this population which we defined as ILC2s, regardless of airway exposure and expression of other surface markers. In the total lung tissue of PBS-treated mice, ~2,500 ILC2s were present, with no significant difference between C57BL/6 or BALB/c mice (Figure 1B), contributing to <1% of all CD45^+^ cells in the lung (Figure S1A). The number of ILC2s in the lungs was increased upon airway exposure to the allergens HDM (~4,500) and ALT (~21,000), although not reaching statistical significance (Figure 1B). However, rIL-33 exposure significantly increased the number of ILC2s in the lung in both mouse strains (~3,10,000) (Figure 1B). A similar trend was identified when analyzing ILC2s as a proportion of CD45^+^ cells; however, the frequency of ILC2s was significantly increased in the lungs of BALB/c mice following HDM treatment and decreased after rIL-33 treatment, compared to C57BL/6 mice (Figure S1A). The total number of ILC2s in the airway [recovered in the Bronchoalveolar lavage (BAL)] following PBS treatment was very low but was increased upon exposure to HDM, ALT, and rIL-33, where rIL-33 again induced the most ILC2s (Figure 1C). Interestingly, following treatment of HDM, ALT, or rIL-33, C57BL/7 mice had an increased number of ILC2s recovered in the BAL, compared to BALB/c mice, although statistical significance was only reached following rIL-33 treatment (Figure 1C). These trends were mirrored when ILC2s were also analyzed as a proportion of CD45^+^ cells (Figure S1B). In the draining mediastinal lymph nodes (mLN), rIL-33 was again the most potent inducer of ILC2s, with a subtle, not statistically significant increases in the total number of ILC2s seen following HDM and ALT (Figure 1D). The same trends were observed when ILC2s were analyzed as a proportion of CD45^+^ cells (Figure S1C). Interestingly, in all pulmonary sites following HDM, ALT, or rIL-33 treatment, the proportion of ILC2s was observed to be lower in BALB/c mice than in C57BL/6 mice (Figures S1D–F), suggesting an expansion of Lineage^−^Gata3^−^ cells. The number of ILC2s identified at each site also correlated with total inflammatory cell influx into the airway (Figures S1G–K). The data points from both replicate experiments demonstrate the reproducibility of the cellular analysis.

### *In vivo* Treatment Alters ILC2 Surface Marker Expression

**Figure 2:**
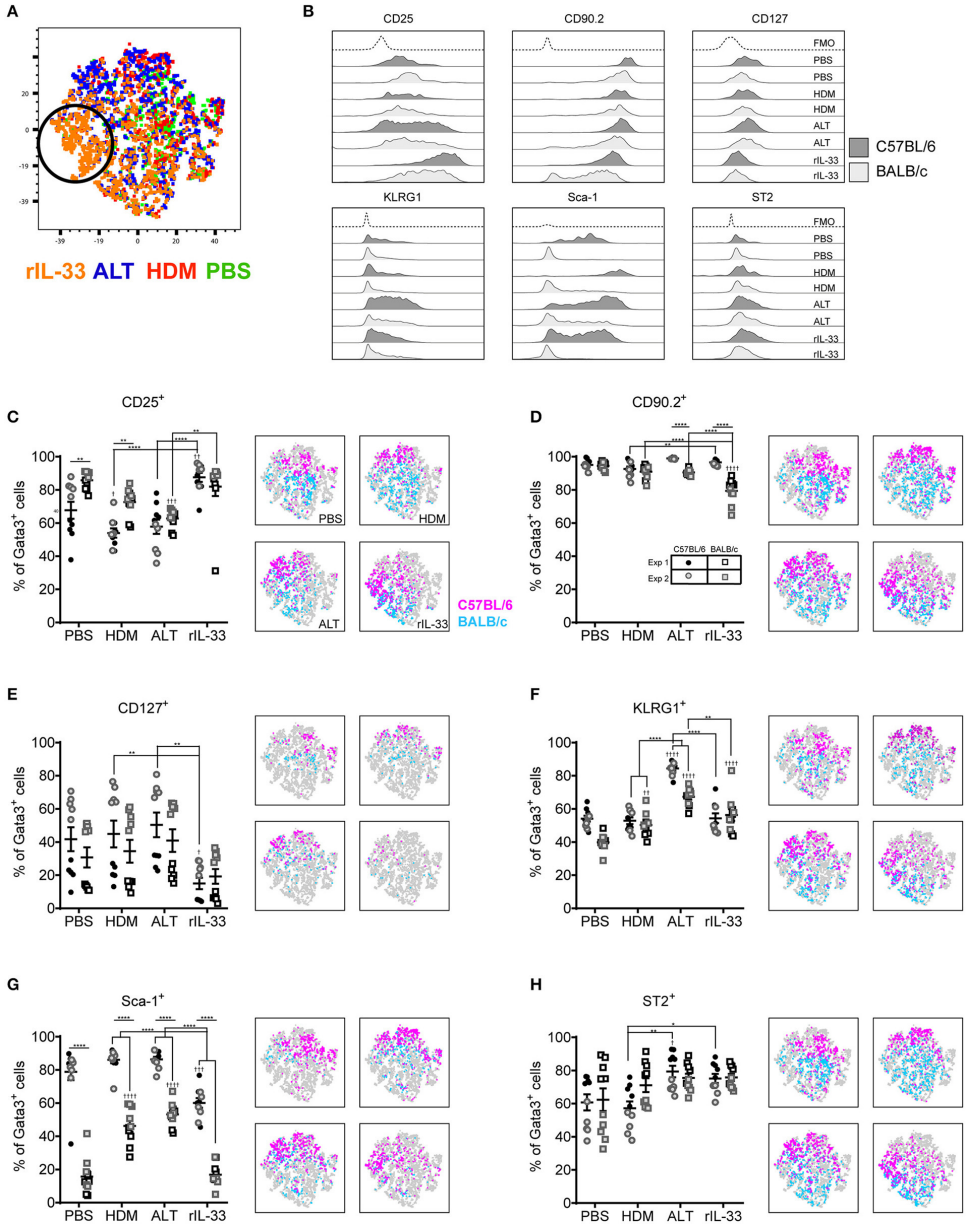
ILC2 marker expression varies between strain and treatment. **(A)** tSNE plot of 4,360 lung Gata3^+^ cells from both C57BL/6 and BALB/c mice. **(B)** Representative histograms for extracellular markers on Gata3^+^ cells taken from C57BL/6 and BALB/c mice treated with PBS, HDM, ALT, or rIL-33. Percentage of Gata3^+^ cells expressing **(C)** CD25, **(D)** CD90, **(E)** CD127, **(F)** KLRG1, **(G)** SCA1, and **(H)** ST2 and accompanying tSNE plots. Data is the combination of two individual experiments, *n* = 10 per group. ^†^*P* < 0.05, ^††^*P* < 0.01, ^†††^*P* < 0.001, ^††††^*P* < 0.0001 compared to PBS control. *P < 0.05, **P < 0.01, ****P < 0.0001 comparing between strains or treatment groups.

We next explored how surface marker expression of pulmonary ILC2s was effected by different airway exposures. Analysis of lung ILC2s via *t*-distributed stochastic neighbor embedding (tSNE) enables a visual representation of cell clustering based on their extracellular marker expression. Using this method we identified a unique cluster of ILC2s from rIL-33 treated mice suggesting a difference in phenotypic marker expression on ILC2s isolated from these mice compared to PBS, HDM and ALT exposed mice (Figure 2A). Therefore, we more closely examined extracellular marker expression on unstimulated ILC2s (no PMA/ionomycin/brefeldin), which had been rested for 4 h at 37°C *ex vivo*, from the lungs of control (PBS), allergen and rIL-33 treated mice. Representative histograms for extracellular markers on ILC2s are shown in Figure 2B. CD25 (IL-2 receptor alpha chain) is reported as a marker of ILC2s and binding of IL-2 by CD25 has been shown to increase expression of IL-13 (17). At baseline (PBS mice), CD25 was expressed on a greater proportion of ILC2s from BALB/c mice (85%) than C57BL/6 mice (65%) (Figure 2C) and a similar trend was observed in mice exposed to HDM. However, in response to ALT or rIL-33 no strain specific effect on expression was observed. The proportion of ILC2s expressing CD25 was reduced in mice exposed to allergen but this was not evident in mice treated with rIL-33, where receptor expression was significantly increased compared to cells recovered from the lungs of allergen exposed mice. Similar patterns of expression were observed on cells isolated from the airways and mLN (Figure S2A).

The immunoglobulin superfamily member CD90.2 was expressed by almost all ILC2s irrespective of strain or challenge suggesting it is a relatively stable marker of ILC2, although there was a small but significant drop in expression in BALB/c mice given ALT or rIL-33 (Figure 2D). The flow cytometry histograms show a clear rightward shift in positive staining (Figure 2B) and the tSNE plots demonstrate that all Lin^−^ Gata3^+^ cells in both strains of mice express this marker (Figure 2D). CD90.2 was expressed by 80% of ILC2s isolated from the BAL of BALB/c mice compared to only 60% of cells recovered from C57BL/6 mice (Figure S2B). Intriguingly, in C57BL/6 mice exposure to either ALT or rIL-33, but not HDM, resulted in an upregulation of CD90.2 on BAL ILC2s to more than 95% of all cells, whereas in BALB/c mice expression was not different from control mice. A similar profile of receptor expression was observed on cells recovered from the mLN (Figure S2B).

CD127 (IL-7Rα) is commonly used as a definitive marker for ILC2. The receptor is utilized by both IL-7 and TSLP and IL-7 signaling is required for the maintenance of IL-7Rα^+^ common lymphoid progenitors that give rise to ILC and other lymphocytes (41). However, surface expression of CD127 was poor on murine ILC2s isolated from the lung tissue, airways or lymph nodes (Figures 2B,E and Figure S2C) and was not reproducible between experiments. Despite using the same clone as reported in the literature (33, 34, 36) there was very little clear positive staining compared to the FMO control. KLRG1 (killer cell lectin-like receptor subfamily G member 1) is a transmembrane protein preferentially expressed on NK cells but has also been utilized as a marker of ILC2s. At steady state KLRG1 is expressed on only 50% of lung ILC2s in C57BL/6 mice and 40% in BALB/c mice (Figure 2F). Surface expression of this marker was dependent on activation of the cells. The strongest inducer was ALT, and expression was also increased in mice exposed to rIL-33. A similar pattern of expression was noted in BAL but in contrast, 90% of the ILC2s recovered from the mLN of mice exposed to rIL-33 expressed KLRG1 (Figure S2D).

In addition to identifying hematopoietic stem cells, Sca-1 is also used as a marker of ILC2s. However, the utility of Sca-1 as a marker of pulmonary ILC2s was particularly strain dependent (Figure 2G). In C57BL/6 mice, Sca-1 was expressed on more than 80% of ILC2s, although this dropped below 60% in response to rIL-33. In contrast <20% of ILC2s from BALB/c mice expressed Sca-1 at baseline (PBS treatment). There was an effect of allergen in that the proportion of Sca-1^+^ cells more than doubled in mice administered HDM and ALT; an effect not seen with rIL-33. Interestingly, for ILC2s recovered from the airway lumen, not the lung tissue, Sca-1 was expressed by almost 60% of cells in both strains of mice and the proportion of Sca-1^+^ cells was not influenced by HDM (Figure S2E). ALT increased expression of Sca-1 in C57BL/6 mice only, whereas rIL-33 elevated expression in C57BL/6 mice but significantly decreased the percentage in BALB/c mice. The IL-33 receptor ST2 has also been used to delineate ILC2 populations. It was expressed by 60% of cells in the lung, increasing to 80% in C57BL/6 mice exposed to ALT or rIL-33 (Figure 2H). A similar profile was observed in BAL and mLN, with the exception of a significant reduction in the proportion of cells expressing ST2 at steady state in C57BL/6 mice (Figure S2F). Thus, CD90.2 was determined to be the most prominent and stably expressed marker on the population of ILC2s in each pulmonary location across all *in vivo* treatments and mouse strains.

### *Ex vivo* Stimulation Effects ILC2 Surface Marker Expression

**Figure 3:**
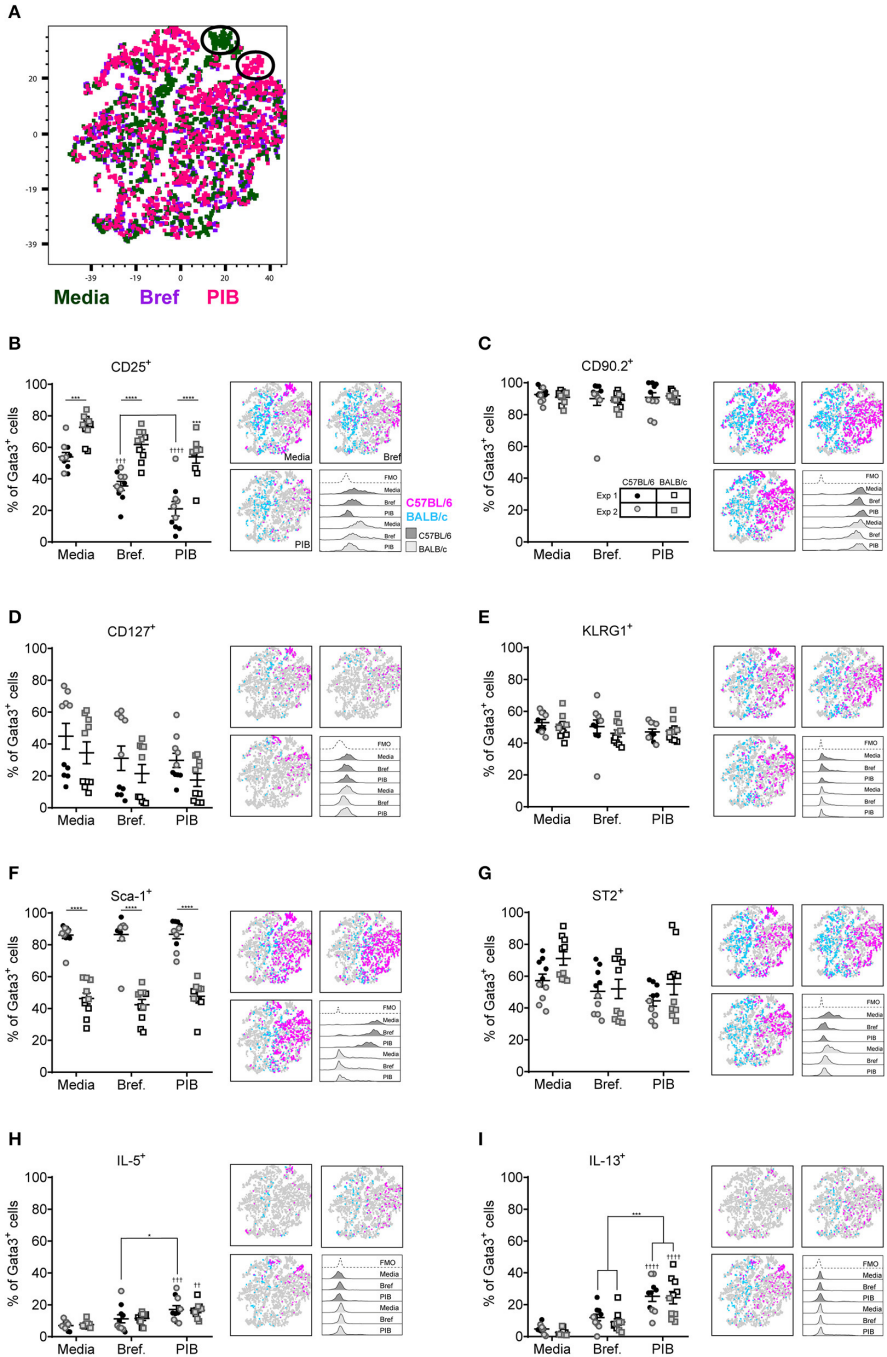
ILC2 marker expression varies with *ex vivo* re-stimulation. **(A)** tSNE plot of 4,437 lung Gata3^+^ cells from both C57BL/6 and BALB/c mice. Representative histograms for extracellular and intracellular markers on Gata3^+^ cells taken from C57BL/6 and BALB/c mice either left in media or stimulated with brefeldin A (Bref) or PMA, ionomycin and Bref (PIB). Percentage of Gata3^+^ cells expressing **(B)** CD25, **(C)** CD90, **(D)** CD127, **(E)** KLRG1, **(F)** SCA1, **(G)** ST2, **(H)** IL-5, and **(I)** IL-13 and accompanying tSNE plots. Data is the combination of two individual experiments, *n* = 10 per group. ^††^*P* < 0.01, ^†††^*P* < 0.001, ^††††^*P* < 0.0001 compared to Media control. *P < 0.05, ***P < 0.001, ****P < 0.0001 comparing between strains or cell stimulations.

Although Gata3 expression defines an ILC2, only those cells secreting type 2 cytokines are functional in terms of driving allergic airways disease. In order to capture cytokine expression by flow cytometry it is necessary to stimulate cells with PMA and ionomycin and utilize a protein transport inhibitor, such as brefeldin, which results in accumulation of cytokine at the Golgi complex. We therefore investigated the effect of incubating total cells *ex vivo* with PMA, ionomycin and brefeldin (PIB), brefeldin alone (Bref) or media control on ILC2 phenotypic markers and cytokine production. We focused those ILC2s induced by HDM as this allergen is clinically relevant [50% of asthmatics allergic to HDM (42)] and is the most commonly used allergen in allergic airway disease models. A tSNE analysis of the extracellular markers and intracellular cytokine staining of ILC2s from a HDM treated mouse demonstrated a number of cells which only expressed specific markers when unstimulated (media only) (Figure 3A). Another unique population of cells was identified when cells were cultured with PIB (Figure 3A). As observed previously, CD25 expression was more prominent on cells isolated from BALB/c mice than C57BL/6 mice (Figure 3B) and expression was reduced following PIB stimulation in either strain. In C57BL/6 mice, only 20% of cells retained cell surface expression of CD25 when stimulated with PIB and the loss of CD25 expression on cells from C57BL/6 mice is clearly visualized on the CD25 tSNE plots (Figure 3B). CD25 expression was similarly downregulated on cells from ALT treated mice (Figure S3A). However, in stark contrast, PIB stimulated cells from rIL-33 treated mice retained expression of CD25, suggesting that CD25 cannot be used to define ILC2 in activated cells from allergen treated mice.

More than 90% of cells from HDM treated mice were CD90.2^+^, irrespective of stimulation (Figure 3C). CD90.2 was also expressed by almost all lung ILC2 from ALT and rIL-33 exposed C57BL/6 mice and, although significantly reduced compared to C57BL/6 mice, CD90.2 was detected on at least 80% of cells from ALT and rIL-33 exposed BALB/c mice even after stimulation (Figure S3B). In HDM treated animals CD127 expression, which we previously identified as a poor marker for ILC2 (Figure 2), was unchanged in the presence of PIB (Figure 3D) and in mice challenged with ALT or rIL-33 surface expression of CD127 was reduced as a result of incubation of the cells with stimulation mix (Figure S3C). KLRG1 was expressed on 50% of cells from HDM and rIL-33 treated mice and its expression was not modulated by PIB (Figure 3E and Figure S3D). Although expression of KLRG1 on cells isolated from ALT treated mice was also not affected by stimulating the cells *ex vivo* there was a strain dependent effect with 80% of cells from C57BL/6 mice expressing this marker compared to 60% of cells from BALB/c mice (Figure S3D). As observed previously, expression of Sca-1 was dependent on strain but was not modulated by *ex vivo* stimulation (Figure 3F and Figure S3E). Likewise, expression of ST2 on ILC2s from HDM and rIL-33 treated mice was not affected by PIB (Figure 3G and Figure S3F). Cells from ALT treated mice were differentially affected and use of brefeldin or cellular activation were associated with a decrease in ST2 expression (Figure S3F).

Expression of IL-5 was only detected when cells were incubated with PIB and between 15 and 20% of Gata3^+^ cells had the capacity to make IL-5 (Figure 3H and Figure S3G). Incubation of lung cells from ALT or rIL-33 treated mice with brefeldin revealed that 30% of the Gata3^+^ cells were making IL-13 *ex vivo* and when cells were incubated with PIB almost 30% of cells from HDM treated mice, 40% of cells from rIL-33 treated mice and 50% of cells from ALT treated mice had the capacity to make IL-13 (Figure 3I and Figure S3H). Interestingly, there were no strain specific effects on the proportions of cells making cytokine.

### Pulmonary Location Influences ILC2 Surface Marker Expression

**Figure 4:**
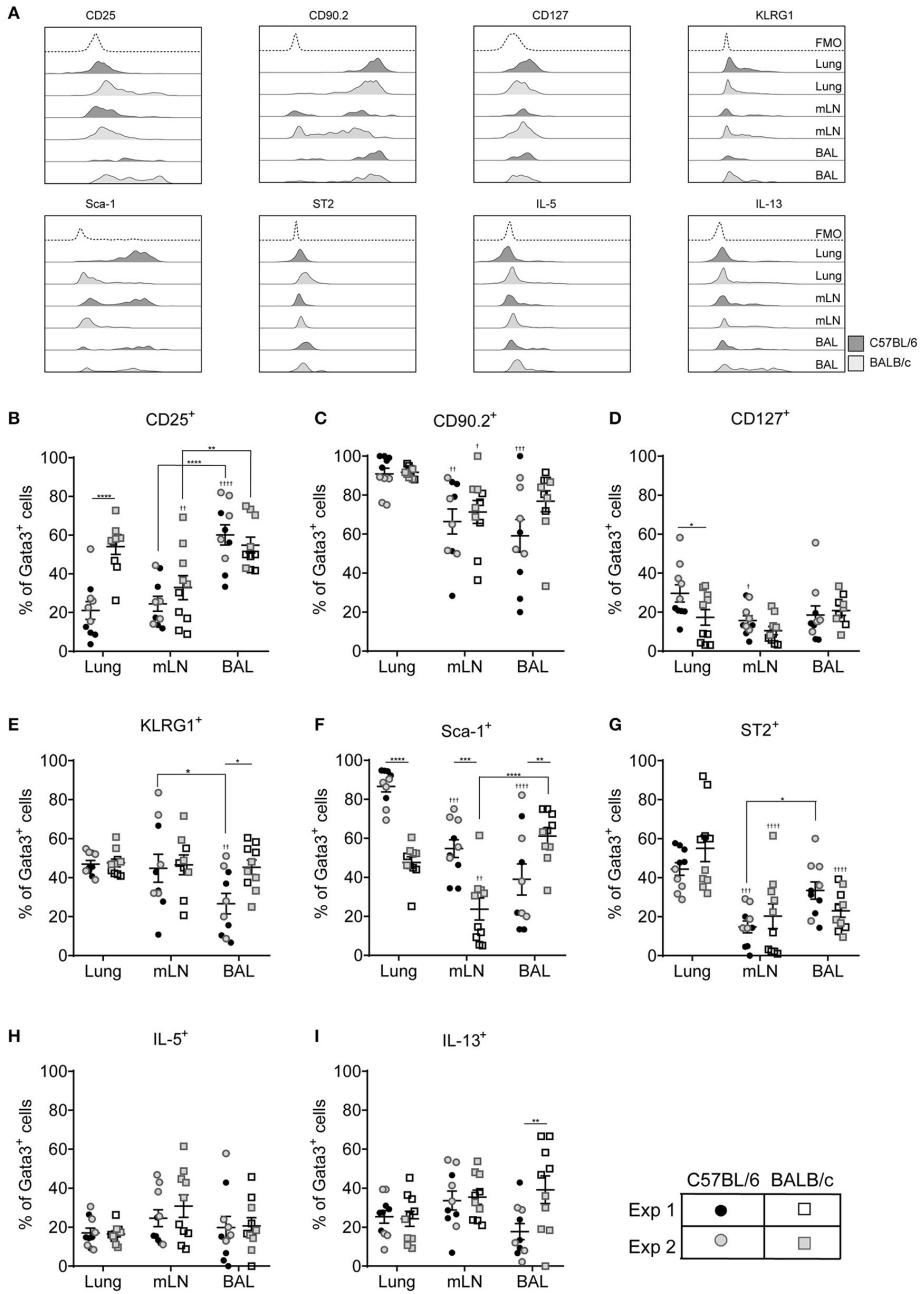
ILC2 marker expression varies between pulmonary locations. **(A)** Representative histograms for extracellular and intracellular markers on Gata3^+^ cells taken from BAL, lung or mLN in C57BL/6 and BALB/c mice. Percentage of Gata3^+^ cells expressing **(B)** CD25, **(C)** CD90, **(D)** CD127, **(E)** KLRG1, **(F)** SCA1, **(G)** ST2, **(H)** IL-5, and **(I)** IL-13. Data is the combination of two individual experiments, *n* = 10 per group. ^†^*P* < 0.05, ^††^*P* < *0.01*, ^†††^*P* < 0.001, ^††††^*P* < 0.0001 compared to lung. *P < *0.05*, **P < *0.01*, ***P < 0.001, ****P < 0.0001 comparing between strains or lung compartment.

We next investigated extracellular marker expression used to phenotype ILC2s based on their anatomical niche in the pulmonary system, comparing cells isolated from the lung tissue with those from the mediastinal lymph nodes (mLN) and airways. Representative histograms for stained cells isolated from the lungs of both C57BL/6 and BALB/c mice exposed to HDM are shown in Figure 4A. In the lung tissue of HDM exposed mice CD25 marked only 20% of cells in C57BL/6 mice but more than 50% of Lin^−^Gata3^+^ cells from BALB/c mice (Figure 4B). In contrast, there was no strain difference in expression of CD25 on cells from the mLN and BAL. However, there was tissue specific expression as CD25 was expressed by 60% of cells from the BAL whereas only half the number of cells from the mLN stained positive for this marker. In comparison, between 20 and 40% of cells isolated from mice administered ALT expressed CD25 whereas 60–85% were CD25^+^ in rIL-33 exposed mice (Figure S4A). CD90.2, which was stably expressed on cells from the lung, was more variable on cells isolated from the airways and mLN of HDM treated mice, particularly in C57BL/6 mice but nonetheless it marked more than 60% of cells (Figure 4C). In contrast CD90.2 was expressed by more than 95% of ILC2s from C57BL/6 mice administered ALT or rIL-33 regardless of the lung compartment the cells were isolated from Figure S4B. In BALB/c mice expression levels of CD90.2 were unaffected by the stimulus used to induce ILC2 with cells originating from the mLN and airways having lower levels of expression than tissue derived cells.

CD127 was not expressed by more than 20% of cells from any lung compartment (Figure 4D) and of those cells expressing this marker CD127 staining showed little separation from fluorescence minus one controls particularly in BALB/c mice (Figure 4A and Figure S4C). Expression of KLRG1 was equivalent in all lung compartments tested in BALB/c mice but only 40% of the cells expressed it (Figure 4E). Expression levels were similar in C57BL/6 mice with the exception of cells recovered from the airways where only 25% of cells had cell surface KLRG1. In mice treated with ALT there was a strain-specific effect in all compartments, with significantly fewer ILC2s from BALB/c mice expressing KLRG1 compared to C57BL/6 mice (Figure S4D). Following rIL-33 treatment, a strain-specific effect on KLRG1 was only seen in the BAL and a pronounced effect of location was observed; 50–60% of ILC2s from the lung and BAL expressing KLRG1 compared to 90% KLRG1^+^ cells recovered from mLN.

Expression of Sca-1 was strain and compartment specific (Figure 4F). Sca-1 is a good marker for ILC2s recovered from the lung tissue in C57BL/6 mice but is expressed on only half of those ILC2s recovered from lungs of BALB/c mice. The same pattern was observed in the mLN, although the proportion of ILC2s expressing Sca-1 is lower than in lung in both strains (55 and 25% in C57BL/6 and BALB/c mice, respectively). Unexpectedly, in the airways the reverse strain specific expression pattern was observed. Sixty percent of ILC2 expressed the marker in BALB/c mice compared to only 35% of cells from C57BL/6 mice. In mice exposed to ALT and rIL-33 again there was a pronounced reduction in the number of ILC2 from BALB/c mice which expressed Sca-1 compared to C57BL/6 in all compartments (Figure S4E). There were no strain specific effects on expression of ST2 which identified only 50% of cells from the lung and between 25 and 15% of Gata3^+^ cells from the airways and mLN, respectively (Figure 4G). Similarly, a lower proportion of cells from the mLN and airways of ALT and rIL-33 treated mice expressed ST2 compared to tissue derived cells (Figure S4F).

ILC2 expression of IL-5 was not dictated by the pulmonary origin of the cells from HDM treated mice (Figure 4H). However, cells from the mLN and airways produced more cytokine than lung derived cells in mice exposed to ALT and rIL-33 (Figure S4G). There was no effect of cellular origin on the capacity of ILC2 to produce IL-13 in mice administered allergen, although ALT is a more potent inducer of IL-13 generation than HDM (Figure 4I and Figure S4H). However, in the rIL-33 model ILC2 recruited to the lungs had a reduced capacity to produce IL-13 (40% of cells) compared to airway and mLN derived cells (more than 60%).

### ILC2 Surface Marker Heterogeneity Is Different in C57BL/6 and BALB/c Mice

**Figure 5:**
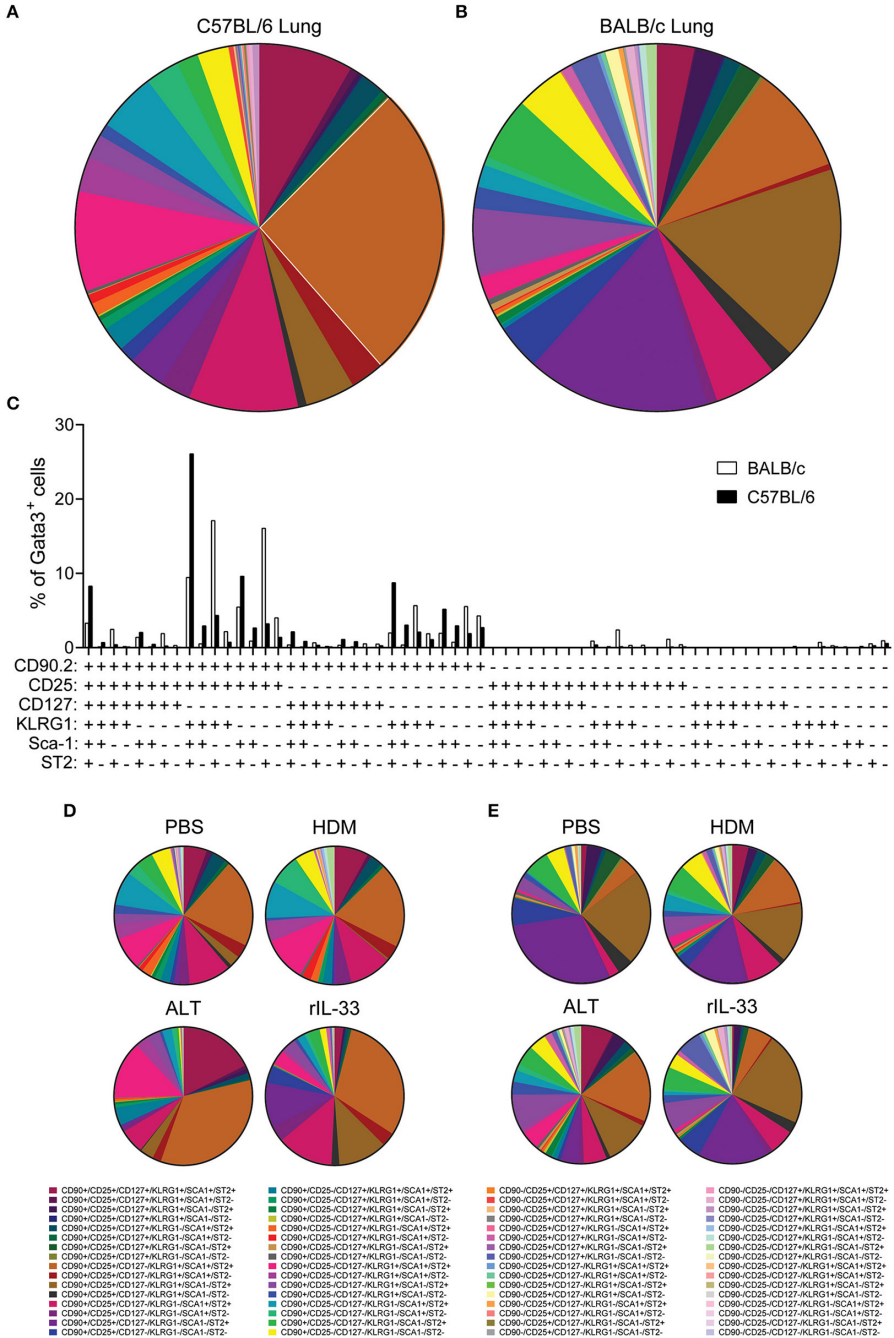
ILC2 surface marker expression combinations are influenced by mouse strain and *in vivo* stimulus. Pie charts depicting the frequency of surface marker combinations present on Lineage^−^Gata3^+^ ILC2s in the lungs of **(A)** C57BL/6 or **(B)** BALB/c mice, average of all *in vivo* treatments. **(C)** Histogram displaying the frequency of surface marker combinations present on Lineage^−^Gata3^+^ ILC2s in the lungs of C57BL/6 and BALB/c mice, average of all *in vivo* treatments. Frequency of surface marker combinations present on Lineage^−^Gata3^+^ ILC2s in the lungs of C57BL/6 **(D)** or BALB/c mice **(E)** following either PBS, HDM, ALT, or rIL-33 *in vivo* treatment. The six surface markers analyzed on ILC2s were CD90.2, CD25, CD127, KLRG1, Sca-1, and ST2.

With pulmonary ILC2 marker expression being significantly different between C57BL/6 and BALB/c mice, we next asked whether there were discrete ILC2 sub-populations in the lungs of the two mouse strains. We therefore identified the frequency of all possible combinations of surface markers on Lin^−^Gata3^+^ ILC2s in the lung of C57BL/6 and BALB/c mice, regardless of *in vivo* treatment with no *ex vivo* stimulation (media only) (Figures 5A,B). With six markers used (CD90.2, CD25, CD127, KLRG1, Sca-1, and ST2) there were 64 possible combinations of surface marker expression. It is of note, that for this analysis we only considered markers as positive (+) or negative (–) and not expression at intermediate or high positive staining, which has been previously used to identify ILC2 sub-populations (18). Analysis of the frequency marker combinations revealed a highly diverse lung ILC2 population in both C57BL/6 and BALB/c mice, with 51 different marker combinations present in C57BL/6 mice and 57 in BALB/c mice. Grossly comparing the two strains of mice, we determined that the frequency of specific marker combinations of lung ILC2s differs between C57BL/6 and BALB/c mice (Figures 5A,B). Furthermore, we identified the most prominent marker combination of ILC2s in each strain; CD90.2^+^CD25^+^CD127^−^KLRG1^+^Sca-1^+^ST2^+^ was the most prevalent surface marker combination in C57BL/6 (26.1% of ILC2s) mice and was over double the frequency of the next most prevalent combination (9.63%). However, in BALB/c mice two different ILC2 marker combinations were equally prevalent in the lung, CD90.2^+^CD25^+^CD127^−^KLRG1^+^Sca-1^−^ST2^+^ (17.12%) and CD90.2^+^CD25^+^CD127^−^KLRG1^−^Sca-1^−^ST2^+^ (16.08%).

We next explored how the different *in vivo* treatments altered the frequency of ILC2 marker combinations in each strain (Figures 5D,E). In C57BL/6 mice, both PBS and HDM treatments produced a remarkably similar repertoire of ILC2 surface marker combinations, whereas ALT and rIL-33 altered the observation marker combinations away from PBS and HDM, specifically expanding the frequency of CD90.2^+^CD25^+^CD127^−^KLRG1^+^Sca-1^+^ST2^+^-expressing ILC2s (Figure 5D). Despite their overall similarity, C57BL/6 lung ILC2 surface marker combination frequency did differ between ALT and rIL-33. Specifically, both CD90.2^+^CD25^−^CD127^−^KLRG1^+^Sca-1^+^ST2^+^ and of CD90.2^+^CD25^+^CD127^+^KLRG1^+^Sca-1^+^ST2^+^ combinations were larger in ALT, whereas of CD90.2^+^CD25^+^CD127^−^KLRG1^−^Sca-1^+^ST2^+^ was increased in rIL-33 (Figure 5D). In Balb/c mice, HDM, ALT, and rIL-33 treatments all altered the frequency of ILC2 marker combinations compared to PBS treatment (Figure 5E). Specifically, HDM and ALT treatment increased the prevalence of CD90.2^+^CD25^+^CD127^−^KLRG1^+^Sca-1^+^ST2^+^-expressing ILC2s in the lungs of Balb/c mice, whereas rIL-33 treatment induced an overall increase in the prevalence of ILC2s not expressing CD90.2 in their surface marker combinations (Figure 5E). Overall, analysis of these six surface markers and their expression combinations on ILC2s from the lungs C57BL/6 and Balb/c mice following different *in vivo* treatments further highlights the heterogeneity of pulmonary ILC2s.

### Sequential Marker Gating Greatly Underestimates Total ILC2 Numbers

**Figure 6:**
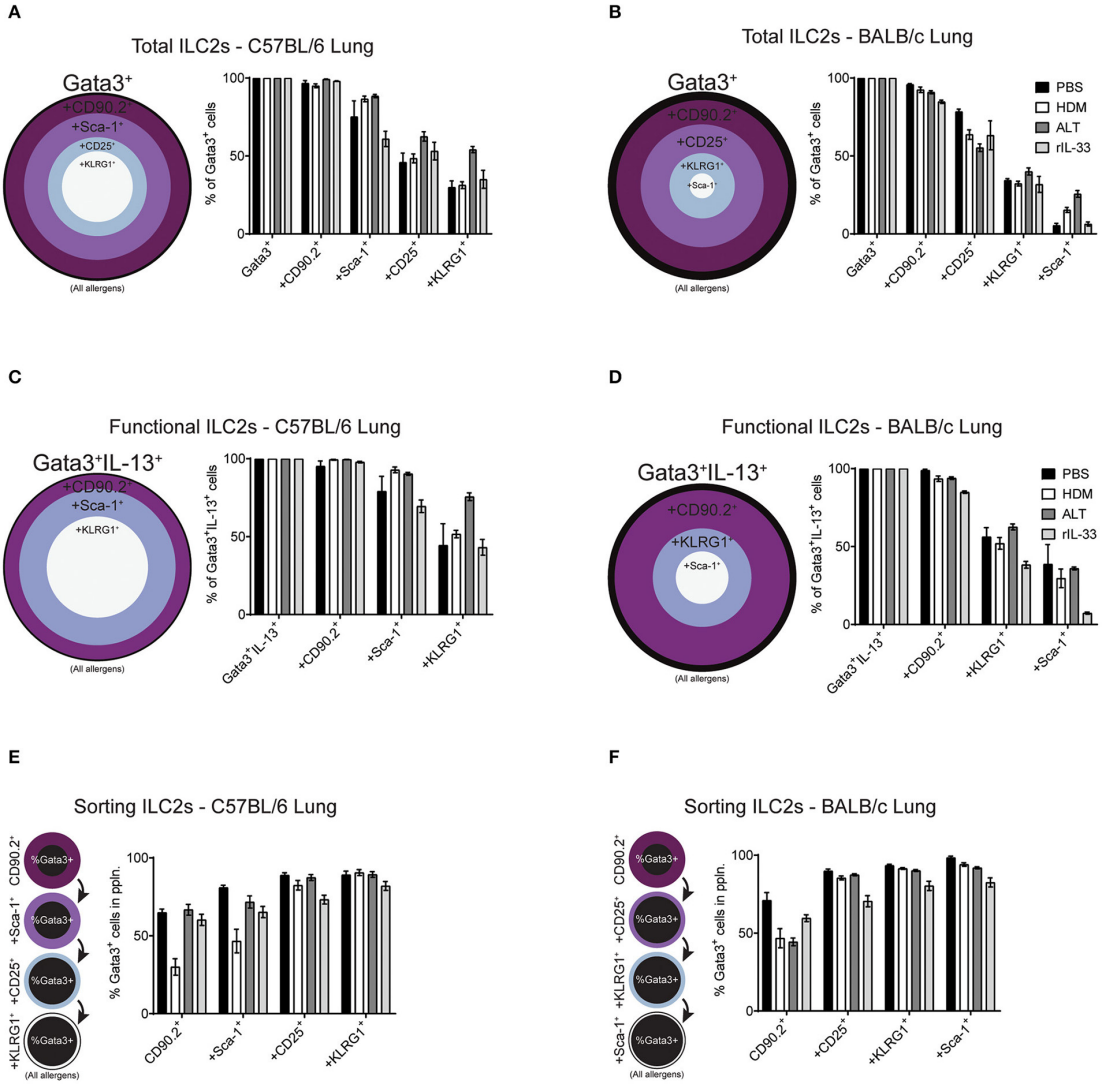
Increasing the number markers used to define ILC2s decreases their perceived frequency. Percentage of Gata3^+^ cells after subsequent gating on extracellular markers, in order of prevalence, in the lungs of C57BL/6 **(A)** or BALB/c mice **(B)**. Percentage of Gata3^+^IL-13^+^ cells after subsequent gating on extracellular markers, in order of prevalence, in the lungs of C57BL/6 **(C)** or BALB/c mice **(D)**. Percentage of Gata3^+^ cells in the population using sequential extracellular markers, in order of prevalence, in the lungs of C57BL/6 **(E)** or BALB/c mice **(F)**.

Our data clearly demonstrates mouse strain-, pulmonary origin, and stimulation-dependent differences when identifying ILC2s in the airways and airway-associated tissue (Figures 1–4). To determine the effect of multiple marker identification of ILC2s in the lung, we performed unbiased sequential gating of appropriate surface markers (excluding CD127 and ST2), of ILC2s (Figure 6). Surface markers were sequentially gated based on their frequency on ILC2s, from highest to lowest (generated for each strain, average of all treatments, media stimulation only).

We first explored the effect of multiple marker sequential gating on the total ILC2 population in the lung of BALB/c and C57BL/6 mice. As determined previously (Figure 2D and Figure S2B), CD90.2 was the most prevalent marker of ILC2s, across all treatments in C57BL/6 (~97%) and BALB/c (~90%) mice (Figures 6A,B). In the lung of C57BL/6 mice, the next most prevalent surface markers were Sca-1, CD25 and then KLRG1, reducing the population size to 78, 53, and 38% of ILC2s, respectively. In BALB/c mice, following CD90.2, the next most prevalent surface markers were CD25, KLRG1, and Sca-1 which, when sequentially gated, reduced the population size to 65, 34, and 13% of ILC2s, respectively. In both strains, there were subtle differences between treatments, although the trends remained similar.

To determine how sequential gating of these markers affects the analysis of functional ILC2s (determined here by their ability to produce IL-13 following PIB stimulation), we again employed the same unbiased approach, based on marker prevalence, but did not use CD25 as we have shown this to be sensitive to *ex vivo* stimulation (Figure 3C). Again, CD90.2 marked the majority of Gata3^+^IL-13^+^ cells in the lungs of C57BL/6 mice (~98%) and BALB/c mice (~93%) (Figures 6C,D). Subsequent gating of Sca-1 in the lungs of C57BL/6 mice or KLRG1 in BALB/c mice resulted in a reduction of Gata3^+^IL-13^+^ cells detected (~83 and ~52%, respectively). Lastly, final gating of KLRG1 in C57BL/6 mice or Sca-1 in BALB/c mice resulted in a reduction of Gata3^+^IL-13^+^ cells detected (~54 and ~28%, respectively). Again, there were subtle differences between allergen treatments in each strain but the trends remained the same.

### Sorting Pulmonary ILC2s From C57BL/6 and BALB/c Mice

When sorting live cells it is not possible to utilize transcription factor staining as this requires cells to be fixed and permeabilized. Therefore, to sort ILC2s Gata3 expression cannot be used, unless a reporter mouse is available. In this case, we used our sequential gating strategies to determine the surface markers that would be required to reliably sort a pure population of Gata3^+^ ILC2s from the lung of both C57BL/6 and BALB/c mice (Figures 6E,F). In C57BL/6 mice, sequentially gating on CD90.2, Sca-1, CD25 and then KLRG1 increased the frequency of Gata3^+^ cells in each population to 55, 66, 83, and 88%, respectively. The same was true for BALB/c mice, where sequential gating of CD90.2, CD25, KLRG1, and then Sca-1 increased the frequency of Gata3^+^ cells to 55, 83, 89, and 92%, respectively.

The order of sequential gating differed in the BAL and mLN for both strains of mice, as the hierarchy of marker prevalence differed between pulmonary sites (Figure 4 and Figure S4). Despite these differences, similar trends in sequential gating of total, functional and sorting ILC2s were seen in the BAL and mLN across all treatments in C57BL/6 and BALB/c mice (Figure S5). Interestingly, when gating “functional ILC2s” in the BAL and mLN of C57BL/6 mice, CD90.2 sharply reduces the frequency of Gata3^+^IL-13^+^ ILC2s identified following PBS or HDM treatment (~30 and 20%, respectively).

### ILC2 Phenotype Identified Using Gata3 Staining Is Comparable to ILC2s Identified With an *Il13*^gfp^ Reporter Mouse

**Figure 7:**
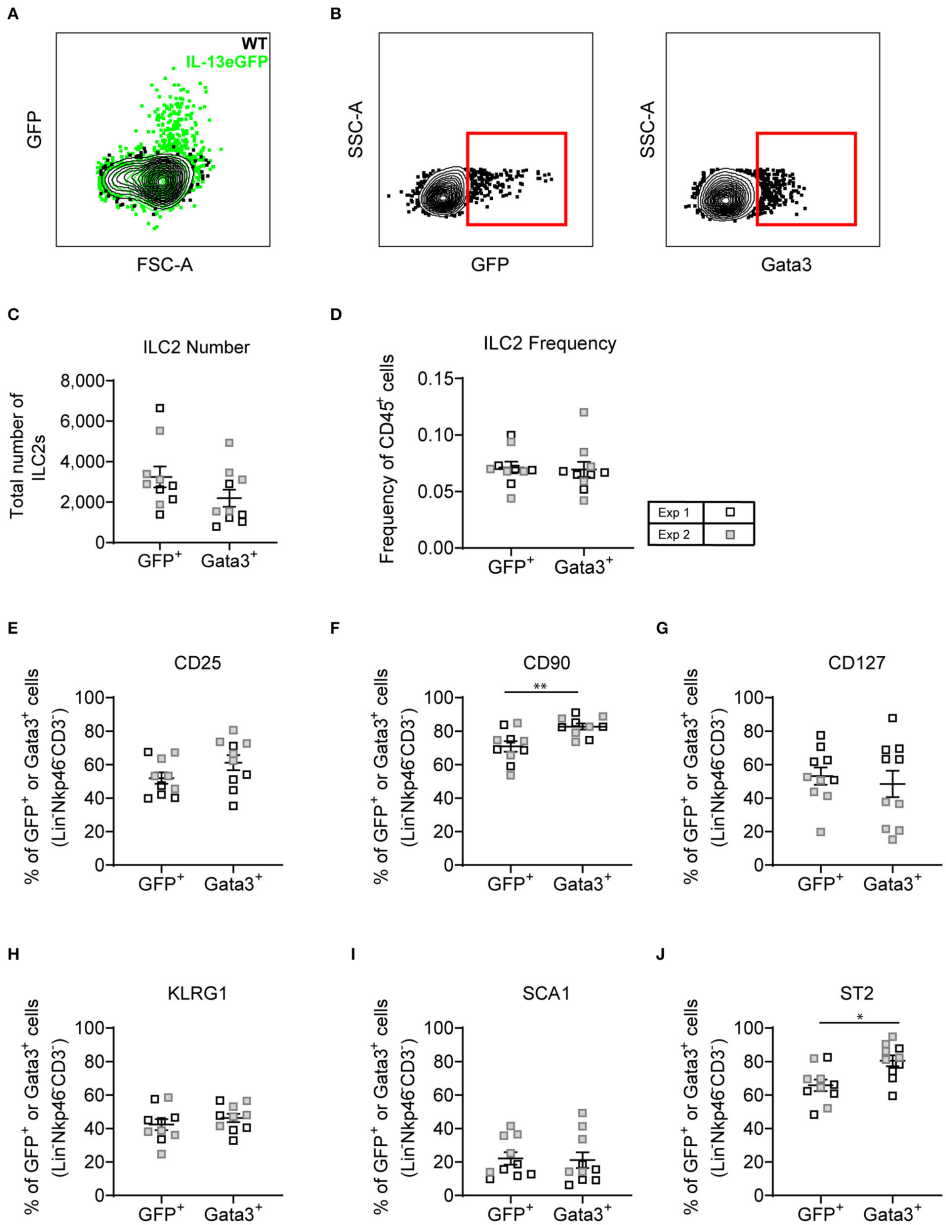
Lineage negative IL-13eGFP^+^ cells show the same extracellular marker expression as Gata3^+^ cells. **(A)** Contour plot depicting the GFP expression of ILC2s in WT and IL-13eGFP mice. **(B)** Gating schematic of *Il13*^gfp+^ and Gata3^+^ ILC2s. Populations are pre-gated on Single cells, Live, CD45+, Lymphoid, Lineage^−^ (TCRβ, TCRγδ, CD3e, CD5, CD19, CD11b, CD11c, FCεR1, GR-1, F4/80, and TER-119 negative), CD3^−^, Nkp46^−^. **(C)** Total number of Lineage^−^ CD3^−^ Nkp46^−^ GFP^+^, or Gata3^+^ cells. **(D)** Frequency of Lineage^−^ CD3^−^ Nkp46^−^ GFP^+^, or Gata3^+^ as a proportion of CD45^+^ cells. Percentage of CD25 **(E)**, CD90.2 **(F)**, CD127 **(G)**, KLRG1 **(H)**, expressing GFP^+^ or Gata3^+^ lung cells. **(F)** Percentage of CD90 expressing GFP^+^ or Gata3^+^ lung cells. **(G)** Percentage of CD127 expressing GFP^+^ or Gata3^+^ lung cells. **(H)** Percentage of KLRG1 expressing GFP^+^ or Gata3^+^ lung cells. **(I)** Percentage of SCA1 expressing GFP^+^ or Gata3^+^ lung cells. **(J)** Percentage of ST2 expressing GFP^+^ or Gata3^+^ lung cells. Data is the combination of two individual experiments, *n* = 10 per group. *P < 0.05, **P < 0.01.

With both transcription factor or type-2 cytokine reporter mice commonly used to identify ILC2s, we asked whether an *Il13*^gfp^ reporter mouse (BALB/c background) yielded similar ILC2 phenotype as utilizing Gata3 intranuclear staining. Firstly, we confirmed that administration of intranasal HDM caused the expression of GFP tagged IL-13 to be produced in the lung in the *Il13*^gfp^ reporter mice (Figure 7A). We next defined our ILC2s as either *Il13*^gfp+^ or Gata3^+^, from Lineage^neg^ cells, and investigated their surface marker expression (Figure 7B). Detection of the *Il13*^gfp^ signal was severely compromised in Gata3-stained cells due to the cell permeabilization required, therefore co-expression of *Il13*^gfp+^ and Gata3^+^ ILC2s could not be determined. Instead, paired samples were treated with or without cell permeabilization allowing for comparison of *Il13*^gfp+^ and Gata3^+^ ILC2s. Analysis showed a small insignificant difference in the total number of ILC2s identified (Figure 7C) and a comparable frequency of CD45^+^ cells (Figure 7D). The frequency of CD25, CD127, KLRG1, and Sca-1 were not significantly different between Gata3^+^ or *Il13*^gfp+^ ILC2s (Figures 7E–J). However, a significant increase in frequency of CD90.2 and ST2 expression on Gata3^+^ ILC2s was observed, compared to *Il13*^gfp+^ ILC2s (Figures 7F,J). No significant differences in surface marker expression between *Il13*^gfp+^ or Gata3^+−^ ILC2s was observed in the BAL (Figure S6A). However, in the mLN the expression of CD25, CD90.2, KLRG1, and ST2 was significantly increased on Gata3^+^ ILC2s, compared to *Il13*^gfp+^ ILC2s (Figure S6B).

## Discussion

Pulmonary ILC2s are important in allergic lung inflammation (25, 26), as well as promoting homeostasis following viral infection (28), and are therefore the subject of investigation across many laboratories worldwide. ILC2s have commonly been identified using surface marker expression. Based on our extensive study phenotyping pulmonary ILC2s in a variety of conditions, we suggest that Gata3 transcription factor staining, after exclusion of lineage-positive cells, should be used to solely define a global population of ILC2s in the lung and airways. This is underlined by our findings that utilizing the most prevalent surface markers underestimated the total Gata3^+^ ILC2 population (Figure 5 and Figure S5). Subsequent surface marker expression therefore serves to describe ILC2 phenotype, although care must be taken as we clearly demonstrate that many surface markers are differentially expressed depending on their environment (mouse strain, allergen treatment or *ex vivo* re-stimulation conditions). Of course, type 2 cytokine or Gata3-reporter mice are helpful tools in identifying and sorting ILC2s, although not essential. This is important as these tools are not always available or convenient to use.

In a recent review it was recognized that ILC2s are not a “uniform population” and that there are “inconsistencies in the markers they express” (43). Natural ILC2 (nILC2) are IL-33 responsive cells whereas IL-25 induces inflammatory ILC2 (iILC2) (18), particularly in the gut, and these cells are capable of migrating to other mucosal sites such as the lung (20). This particular subset of ILC2 express the activation marker KLRG1 but do not express ST2 which precludes its usefulness as a definitive marker of ILC2 populations (44). In addition, we demonstrate that using ST2 to define ILC2s is problematic due to instability of expression across different sampling locations, allergen-specific alterations, and downregulation upon *ex vivo* re-stimulation. Detailed single cell analysis suggests that there may be up to 15 different subcategories of ILC (43). Our surface marker phenotyping of Gata3^+^ ILC2s somewhat supports these transcriptional findings, although tSNE analysis of protein expression did not uncover clearly distinct sub-categories of ILC2s, but instead overlapping populations which do not uniquely express a group of surface markers. This observation was supported when we analyzed lung ILC2 surface marker combinations and demonstrated a multitude of ILC2 surface marker “flavors” with no discrete combination exceeding 34% of Gata3^+^ ILC2s. It must be noted that we have only looked at the six most commonly reported surface proteins due to limitations of flow cytometry. Perhaps more detailed analysis of pulmonary ILC2 surface marker expression by CyTOF would further uncover distinct ILC2 sub-populations, akin to that described by single cell transcriptional analysis. Transcriptome analysis of ILC2s elicited by rIL-33 isolated from the BAL and mLN of mice revealed tissue-specific gene expression signatures in ILC2s, perhaps responsible for the subtle differences we observed in surface marker expression between these two sites [Figure 4; (45)]. This tissue-specific transcriptome is supported when comparing the transcriptome of ILC2s from different mucosal sites (12). However, paired transcriptome and epigenome analysis of ILC2s from the BAL and mLN suggest highly comparable epigenomic profiles despite differential gene expression (45), suggesting tissue-specific transcriptomes arise from the same epigenome. With the advances in multiparameter flow cytometry and integration with transcriptome and epigenome analysis (such as CITE-seq), It would be of great interest to determine the interaction between epigenome, transcriptome and protein expression in ILC2s from different sites and following different stimulations.

Interestingly, it has recently been identified that sex-specific ILC2 populations arise in unchallenged mice at 12 weeks of age. Specifically, pulmonary ILC2s from males were predominantly KLRG1^+^ whereas pulmonary ILC2s from female mice uniquely have a prominent population of functional KLRG1^−^ ILC2s (46). In our study we used female mice for our analysis, but we did observe the same female-specific KLRG1 phenotype (~50% of ILC2s are KLRG1^−^ in the lungs of C57BL/6 mice). It would be of interest to further identify sex-specific differences in ILC2 phenotype during allergen challenge.

Macro- and micro-environmental cues are likely to contribute to the heterogeneity observed in the ILC2 population. The importance of these environmental cues in dictating ILC2 phenotype is highlighted by the different surface marker expression of ILCs in mice treated with HDM, ALT, or rIL-33—all of which induce “type 2” inflammation in the lung. Tissue-specific signals have been demonstrated to imprint and prime ILC2s for function in said tissue, such as lung ILC2s expressing *Il1rl1* (IL-33 receptor) and gut ILC2s expressing *Il17rb* (IL-25 receptor) (12). Recently, Huang et al. identified an ILC2 sub-population termed “inflammatory ILC2s” (iILC2s) in the peritoneal cavity through differential expression of ST2 and KLRG1, induced by IL-25 only and not IL-33 treatment (18). We therefore looked in our models for these iILC2s. As predicted, rIL-33 intranasal administration induced a similar ST2 and KLRG1 expression profile to intraperitoneal administration of rIL-33, and not that of rIL-25, demonstrated by Huang et al. In addition, the clinically relevant allergens, HDM or ALT, also failed to illicit the IL-25-dependent iILC2 cells in the lung (data not shown). Our data therefore corroborates the consensus that ILC2 populations take their cues from the surrounding environment, and, specifically in the lung, ILC2 activation is predominantly mediated by IL-33 (12). It would be interesting to compare the effect of recombinant IL-33, IL-25, and TSLP administration on pulmonary ILC2 phenotype to further explore tissue-specific ILC2 activation signals.

A critical observation from our study is the considerable difference in ILC2 surface marker expression observed between rIL-33 treatment and the two clinically-relevant allergens, HDM and ALT, in both strains of mice. Recombinant IL-33 is commonly used to study pulmonary ILC2s since, as we demonstrate, administration to the lungs induces a supraphysiological increase in number of this rare cell population. However, it is questionable whether it is wise to draw conclusions from these phenotypically different, supraphysiological ILC2s and apply them to disease scenarios. Caution must be exercised when extrapolating findings from rIL-33-induced ILC2 biology into physiological settings because, as we demonstrate, rIL-33 generates a distinct ILC2 population, in terms of cytokine production and surface marker phenotype. This population is unlike anything observed when using clinically relevant allergens, with one dose of ALT (10 μg) demonstrated to induce a 3 log lower concentration of IL-33 than one dose of rIL-33 (1 μg) (47). The difference observed in surface marker expression suggests that they might also differ functionally from those in a disease environment. It would therefore be beneficial for future research to compare observations of rIL-33-induced ILC2 biology with physiologically-relevant ILC2s.

In highlighting the considerable heterogeneity of pulmonary ILC2s in different models of allergic airway inflammation, our analysis only provides a snapshot of ILC2 phenotype. Unsurprisingly, other studies have identified dynamic changes of ILC2 surface marker expression following stimulation over time. For example, ILC2 expression of CD25 is dynamically regulated following influenza virus infection, with expression peaking at 7 days post-infection with subsequent downregulation over time (38). It would therefore be of interest to investigate the phenotype of ILC2s in models of allergic airway inflammation during the early initiation of inflammation, throughout chronic inflammation and during the repair and resolution phase following cessation of allergen treatment.

Enzymatic tissue digestion has previously been demonstrated to alter surface marker expression frequencies on a variety of cells (48–50). In our analysis we utilized collagenase and DNase digestion protocol to obtain cells from the lung, a method optimized for obtaining pulmonary leukocytes from models of allergic airway inflammation. We cannot conclude any effects of enzymatic digestion on ILC2 surface marker expression from this study and is therefore a caveat which must be considered, particularly when comparing ILC2 surface marker expression between those obtained from the lung with ILC2s from the mLN and BAL.

As previously highlighted, pulmonary ILC2s are important drivers of type 2 inflammation and immunity and have been linked to the promotion of allergy and asthma. As we propose, these cells are identified by their expression of Gata3 and the type 2 cytokine IL-13. However, ILC2 plasticity is increasingly being recognized, with ILC1/2s also co-expressing “type 2” genes and genes associated with ILC1s, whilst ILC2/3 also encode genes characteristic of ILC3s, at least in the gut (51). Such studies investigating ILC1/2/3 plasticity in the lung are currently lacking and are a limitation of the study displayed here. Evidence suggests that ILC dynamic phenotypes are a reflection of the altered inflammatory landscape that occurs in implementing immune responses and that these may be subtly different in diverse strains of mice. The data from this study demonstrates that ILC2s are well-adapted to respond to different environmental stimuli and their own micro-environmental niche as receptor expression patterns are dictated by location, in addition to initiating stimuli. There is considerable heterogeneity in the phenotype of CD45^+^Lin^−^ Gata3^+^ ILC2s. Cells respond differentially to micro-environmental changes within the lung tissue, at the mucosal surface of the airways and in the lung draining lymph nodes, likely a consequence of fluctuating stimuli and the prevailing cytokine milieu. Thus, although the ILC1/2/3 paradigm has provided a useful framework for understanding ILC biology, the capacity for ILCs to respond to different cues in different settings is complex and more specific criteria are required to define the plethora of ILC activation states that occur *in vivo*, particularly during disease. Identifying potential co-expression of ILC1,−2, and−3 master transcription factors and associated surface marker expression in the lung following clinically relevant allergen treatment would be of interest.

As a community of scientific researchers, and also of peer reviewers, it is important that we appreciate the flexible nature of ILC2s; a lineage negative cell expressing CD45 and Gata3 defines an ILC2 and we should not insist on multiple additional specific markers to define them as their expression is subject to mouse strain, inducing stimulus, and tissue residency. With our ever-increasing understanding of ILC2 biology, perhaps we should employ a similar method to define them as we do Th2 cells; TCRαβ-, CD4- and Gata3-positive (CD45-positive, lineage-negative and Gata3-postive for ILC2s), with other surface markers used to phenotype and cytokine production for function, according to the specifics of the particular environmental milieu.

## Data Availability Statement

The raw data supporting the conclusions of this article will be made available by the authors, without undue reservation, to any qualified researcher.

## Ethics Statement

This animal study was reviewed and approved by Imperial College's Animal Welfare and Ethical Review Body (AWERB). All procedures were conducted in accordance with the Animals Scientific Procedures Act (ASPA) 1986.

## Author Contributions

LE, LG, RO, WB, and FP performed the experiments. LE and RO performed the analysis of the data and statistics. LE and LG wrote the manuscript. LG conceived the idea for the study. CL discussed the data and edited the manuscript.

### Conflict of Interest

The authors declare that the research was conducted in the absence of any commercial or financial relationships that could be construed as a potential conflict of interest.
