# Supplementary material for: Pulmonary Group 2 Innate Lymphoid Cell Phenotype Is Context Specific: Determining the Effect of Strain, Location, and Stimuli
Source: Front Immunol. 2020 Jan 22;10:3114. doi: 10.3389/fimmu.2019.03114 (PMC6987460; doi:10.3389/fimmu.2019.03114)
Supplement: Supplementary file 1 [file Data_Sheet_1.PDF]

Figure S1

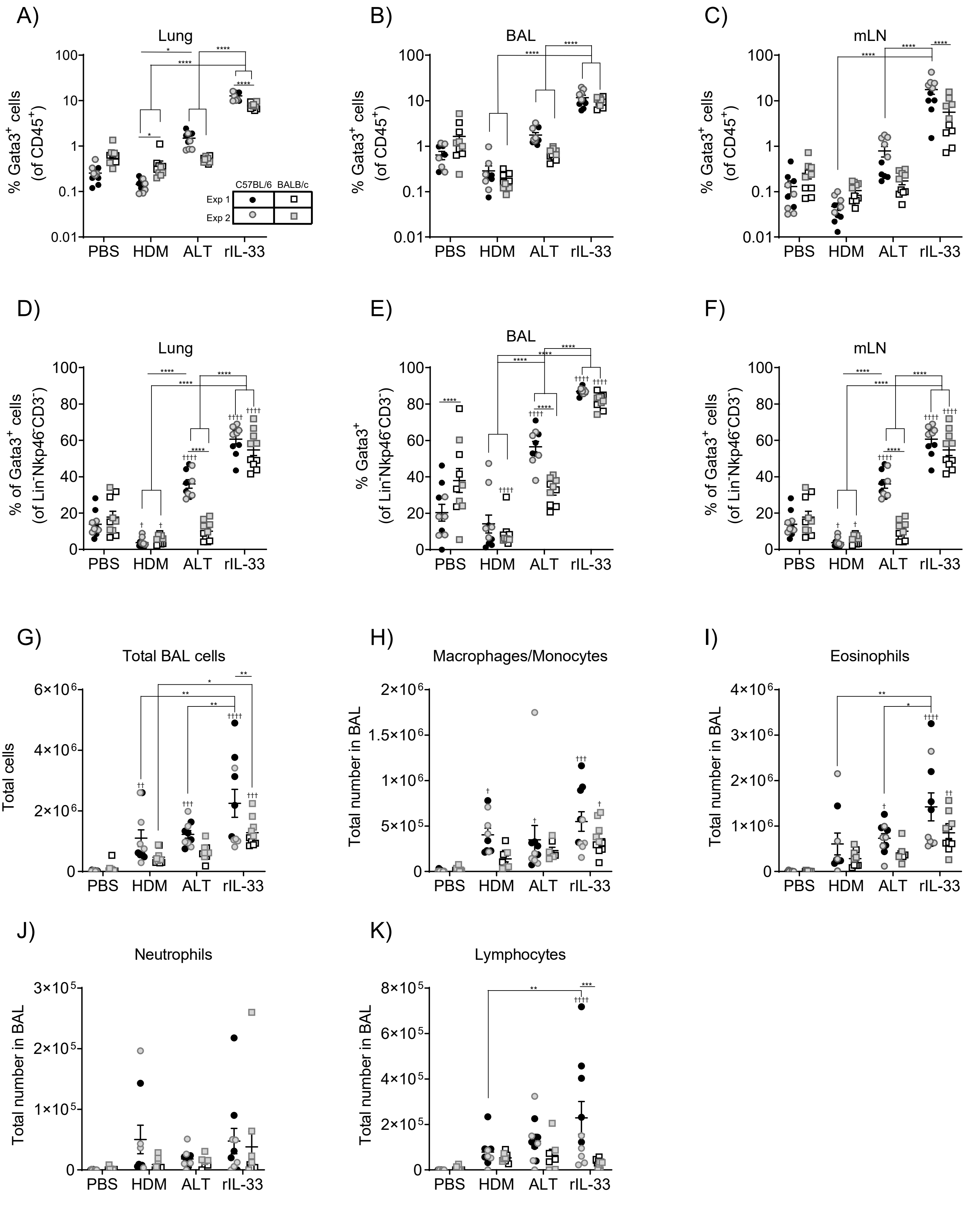
A)
Lung
% Gata3+ cells (of CD45+)
C57BL/6
BALB/c
Exp 1
Exp 2
B)
BAL
% Gata3+ cells (of CD45+)
C)
mLN
% Gata3+ cells (of CD45+)
D)
Lung
% of Gata3+ cells (of Lin-Nkp46-CD3-)
E)
BAL
% Gata3+ (of Lin-Nkp46-CD3-)
F)
mLN
% of Gata3+ cells (of Lin-Nkp46-CD3-)
G)
Total BAL cells
Total cells
H)
Macrophages/Monocytes
Total number in BAL
I)
Eosinophils
Total number in BAL
J)
Neutrophils
Total number in BAL
K)
Lymphocytes
Total number in BAL
PBS
HDM
ALT
rIL-33

Figure S2

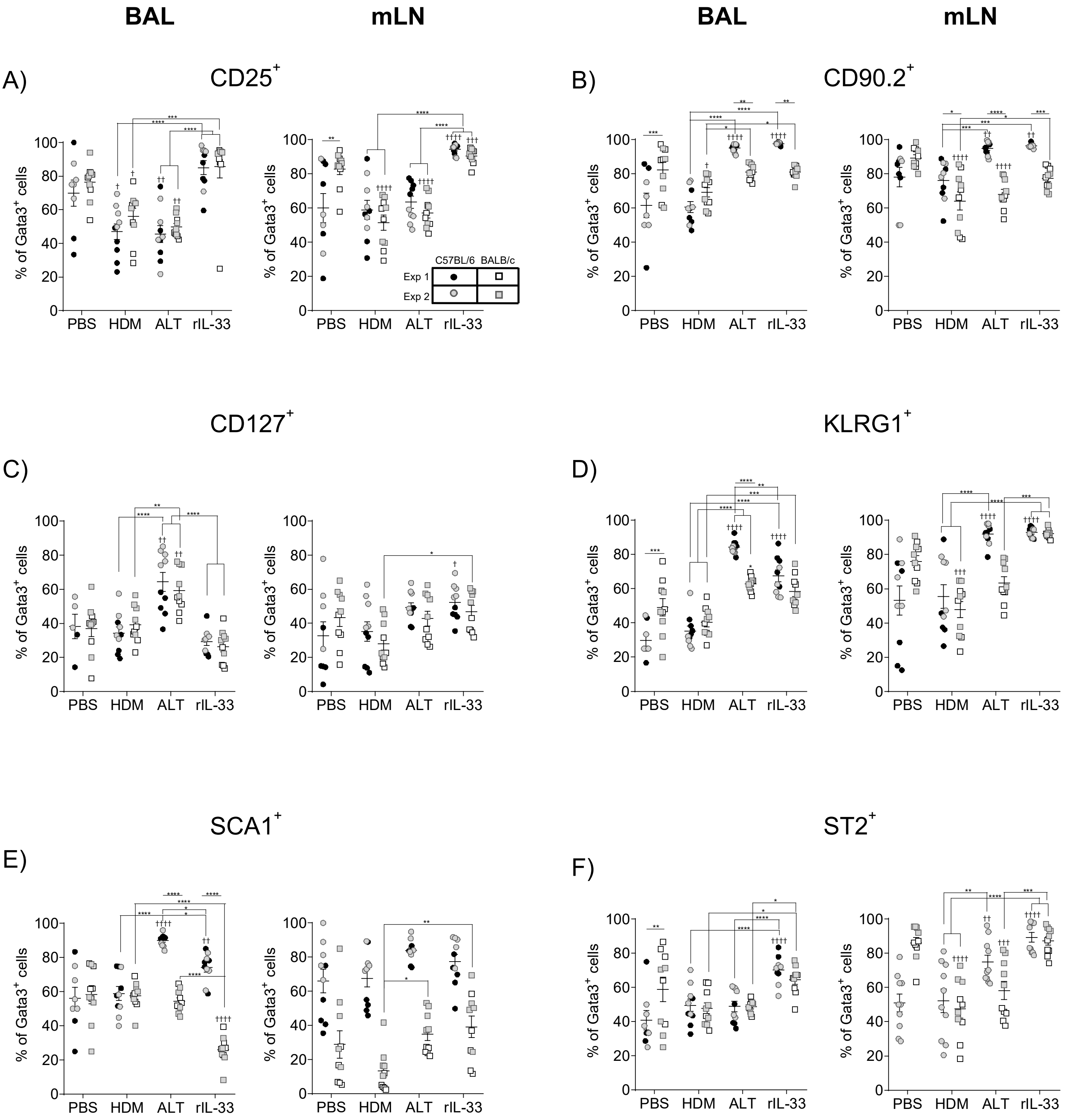

BAL
mLN
BAL
mLN
A)
CD25+
B)
CD90.2+
C)
CD127+
D)
KLRG1+
E)
SCA1+
F)
ST2+
% of Gata3+ cells
PBS
HDM
ALT
rIL-33
C57BL/6
BALB/c
Exp 1
Exp 2

Figure S3

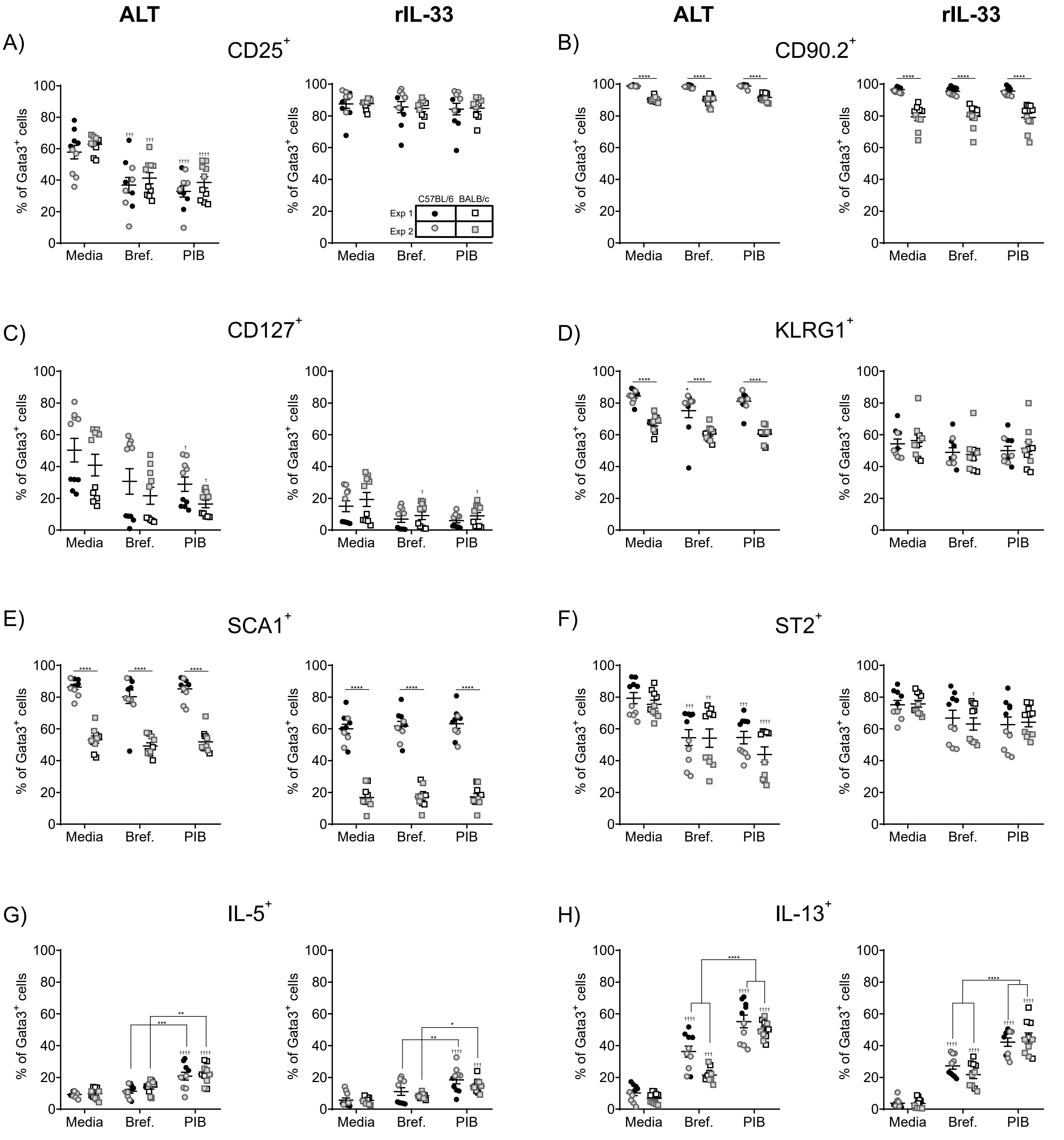

ALT
rIL-33
ALT
rIL-33
A)
CD25+
B)
CD90.2+
C)
CD127+
D)
KLRG1+
E)
SCA1+
F)
ST2+
G)
IL-5+
H)
IL-13+
% of Gata3+ cells
Media
Bref.
PIB
C57BL/6
BALB/c
Exp 1
Exp 2

Figure S4

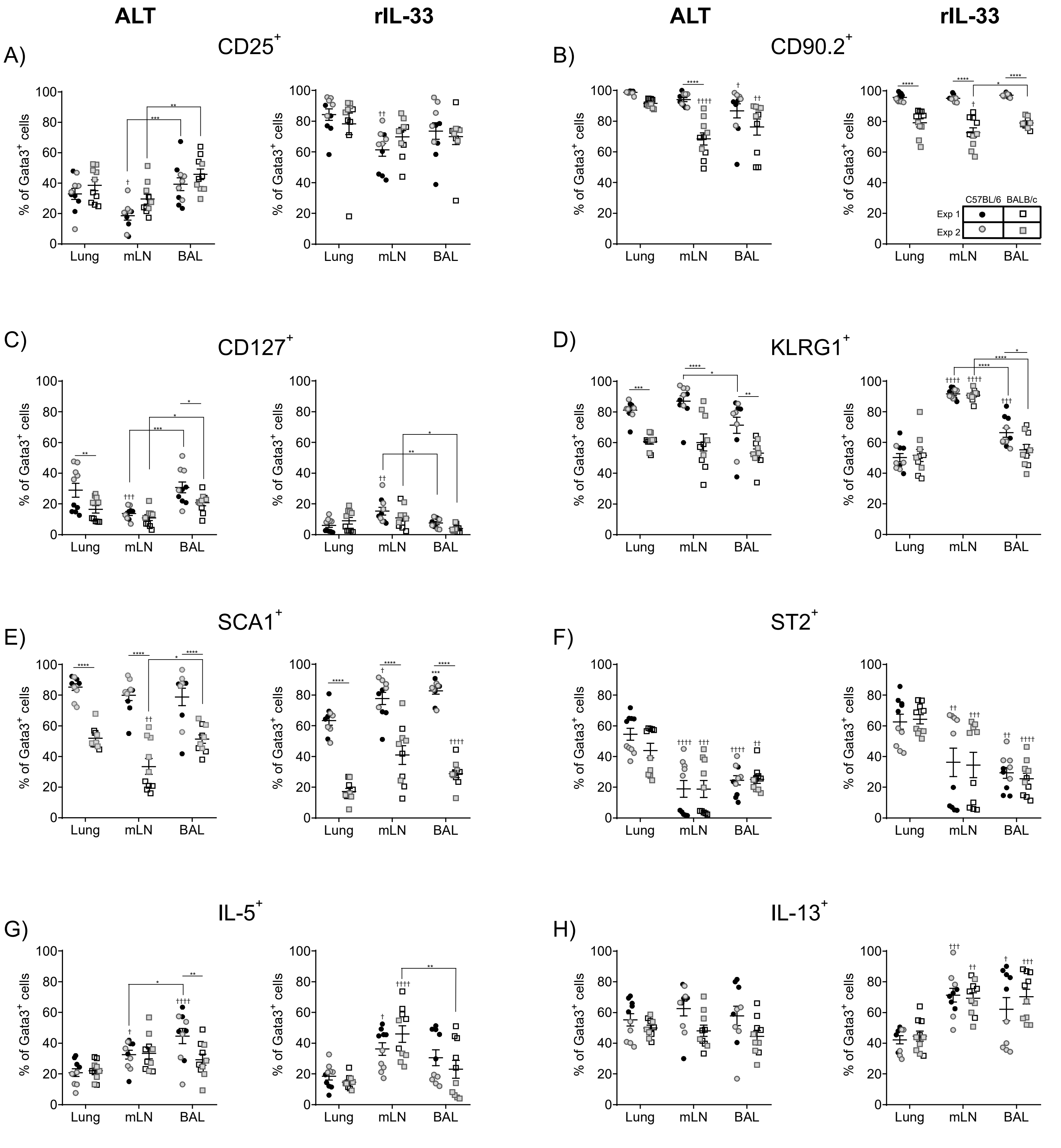

ALT
rIL-33
ALT
rIL-33
A)
CD25+
B)
CD90.2+
C)
CD127+
D)
KLRG1+
E)
SCA1+
F)
ST2+
G)
IL-5+
H)
IL-13+
% of Gata3+ cells
Lung
mLN
BAL
C57BL/6
BALB/c
Exp 1
Exp 2

Figure S5

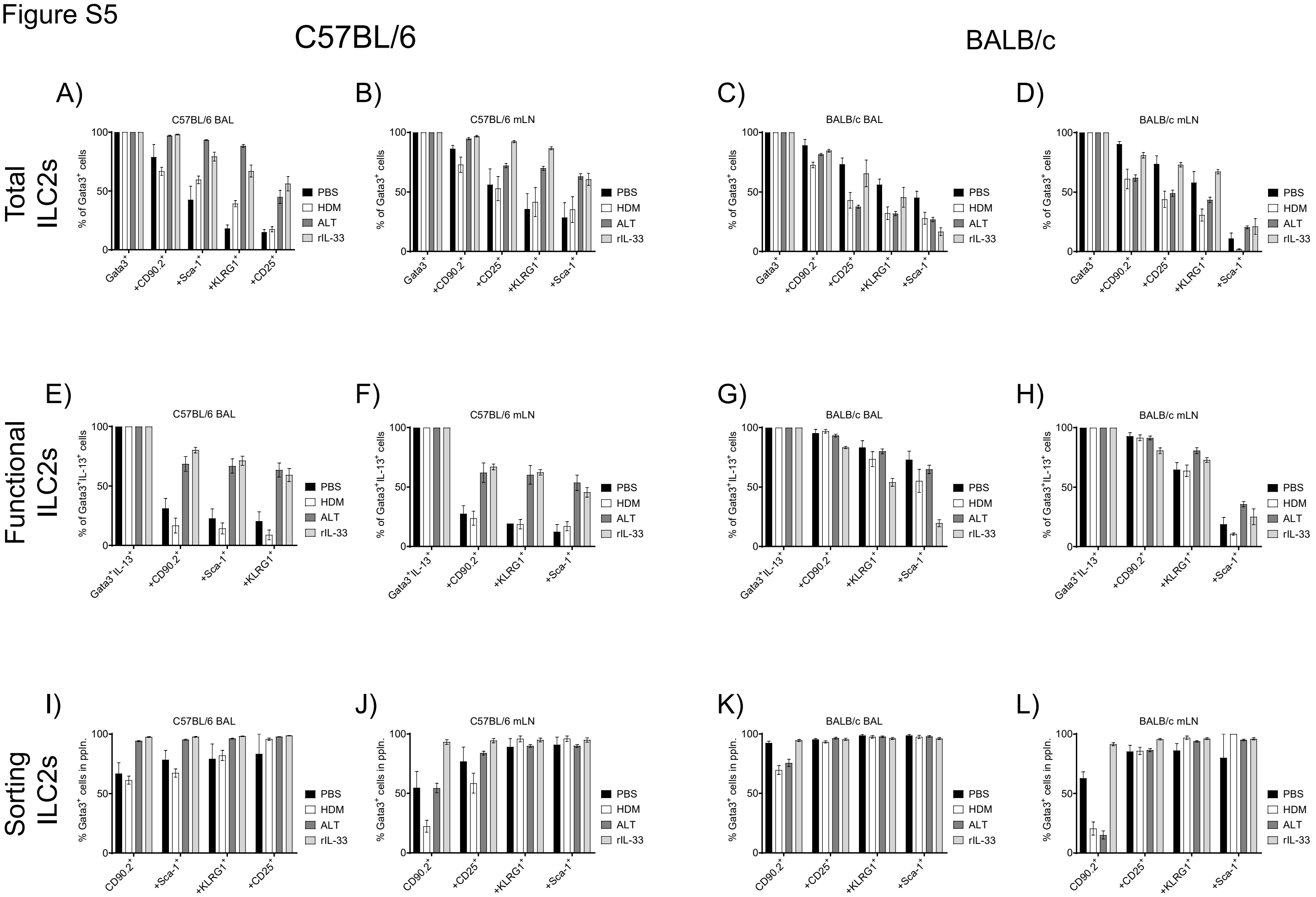
C57BL/6
BALB/c
Total ILC2s
Functional ILC2s
Sorting ILC2s
A)
C57BL/6 BAL
B)
C57BL/6 mLN
C)
BALB/c BAL
D)
BALB/c mLN
E)
C57BL/6 BAL
F)
C57BL/6 mLN
G)
BALB/c BAL
H)
BALB/c mLN
I)
C57BL/6 BAL
J)
C57BL/6 mLN
K)
BALB/c BAL
L)
BALB/c mLN
% of Gata3⁺ cells
% of Gata3⁺IL-13⁺ cells
% Gata3⁺ cells in ppln.
PBS
HDM
ALT
rIL-33
Gata3⁺
+CD90.2⁺
+Sca-1⁺
+KLRG1⁺
+CD25⁺
Gata3⁺IL-13⁺
CD90.2⁺
100
50
0

Figure S6

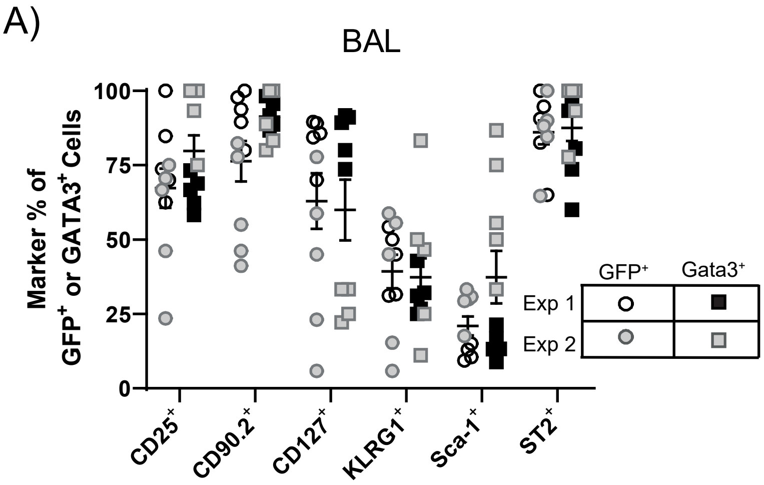
A)
BAL
Marker % of GFP+ or GATA3+ Cells
100
75
50
25
0
CD25+
CD90.2+
CD127+
KLRG1+
Sca-1+
ST2+
GFP+
Gata3+
Exp 1
Exp 2

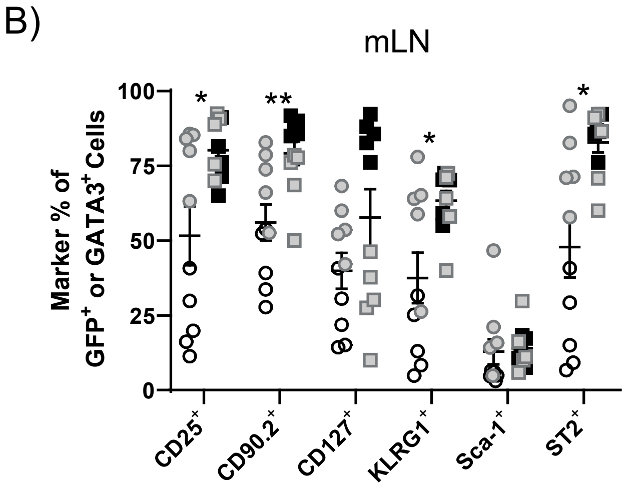
B)
mLN
Marker % of GFP+ or GATA3+ Cells
100
75
50
25
0
*
**
*
*
CD25+
CD90.2+
CD127+
KLRG1+
Sca-1+
ST2+

Fig. S1. Pulmonary ILC2s increase in response to allergen and rIL-33.

The frequency of $Lineage^{-}CD3^{-}NKp46^{-}$ $Gata3^{+}$ cells as a percentage of $CD45^{+}$ cells in the lung (**A**), BAL (**B**) and Mediastinal lymph node (mLN) (**C**). The frequency of $Gata3^{+}$ cells as a percentage of $Lineage^{-}CD3^{-}NKp46^{-}$ cells in the lung (**D**), BAL (**E**) and mLN (**F**). (**G**) The total number of cells present in the BAL. The total number of (**H**) macrophages/ monocytes, (**I**) Eosinophils, (**J**) Neutrophils and (**K**) lymphocytes present in the BAL Data is the combination of two individual experiments. † P <0.05, †† P <0.01, ††† P <0.001, †††† P <0.0001 compared to PBS control. * P <0.05, ** P <0.01, *** P<0.001, **** P <0.0001 comparing between strains or treatment groups.

Fig. S2. ILC2 marker expression varies between strain and treatment in the BAL and mLN.

Percentage of $Gata3^{+}$ cells expressing (**A**) CD25, (**B**) CD90.2, (**C**) CD127, (**D**) KLRG1, (**E**) SCA1 and (**F**) ST2 from the BAL and mLN of C57BL/6 and BALB/c mice treated with PBS, HDM, ALT or rIL-33. Data is the combination of two individual experiments. † P <0.05, †† P <0.01, ††† P <0.001, †††† P <0.0001 compared to PBS control. *P <0.05, **P <0.01, ***P<0.001, **** P <0.0001 comparing between strains or treatment groups.

Fig. S3. ILC2 marker expression varies with *ex vivo* re-stimulation.

Percentage of $Gata3^{+}$ cells expressing (**A**) CD25, (**B**) CD90.2, (**C**) CD127, (**D**) KLRG1, (**E**) SCA1, (**F**) ST2, (**G**) IL-5 and (**H**) IL-13 from the lungs of C57BL/6 and BALB/c mice treated with ALT or rIL-33 and either left in media or stimulated with brefeldin A (Bref) or PMA, ionomycin and Bref (PIB) *ex vivo*. Data is the combination of two individual experiments. †† P <0.01, ††† P <0.001, †††† P <0.0001 compared to Media control. *P <0.05, **P<0.01, ***P <0.001, **** P <0.0001 comparing between strains or cell stimulations.

Fig. S4. ILC2 marker expression varies between pulmonary locations.

Percentage of $Gata3^{+}$ cells expressing (**A**) CD25, (**B**) CD90.2, (**C**) CD127, (**D**) KLRG1, (**E**) SCA1, (**F**) ST2, (**G**) IL-5 and (**H**) IL-13 from the lungs, mLN or BAL of C57BL/6 and BALB/c mice stimulated *ex vivo* with PMA, ionomycin and Bref (PIB) *ex vivo* following ALT or rIL-33 treatment. † P <0.05, †† *P <0.01*, ††† P <0.001, †††† P <0.0001 compared to lung. *P <0.05, **P <0.01, ***P <0.001, **** P <0.0001 comparing between strains or lung compartment.

Fig. S5. Increasing the number markers used to define ILC2s decreases their perceived frequency in the BAL and mLN.

The effect of sequential surface marker gating, in order of prevalence, on the frequency of $Lineage^{-}Gata3^{+}$ ILC2s following either PBS, HDM, ALT or rIL-33 *in vivo* treatment in the BAL (**A**) and mLN (**B**) of C57BL/6 mice or BAL (**C**) and mLN (**D**) of BALB/c mice.

The effect of sequential surface marker gating, in order of prevalence, on the frequency of Lineage^{-}Gata3^{+}IL-13^{+} ILC2s following either PBS, HDM, ALT or rIL-33 *in vivo* treatment in the BAL (**E**) and mLN (**F**) of C57BL/6 mice or BAL (**G**) and mLN (**H**) of BALB/c mice.
The frequency of Gata3^{+} ILC2s present following sequential marker gating in mice treated with either PBS, HDM, ALT or rIL-33 in the BAL (**I**) and mLN (**J**) of C57BL/6 mice or BAL (**K**) and mLN (**L**) of BALB/c mice.

Fig. S6. Lineage-negative GFP^{+} cells display a similar phenotype as Lineage-negative Gata3^{+} cells.

The frequency of extracellular marker expression on ILC2s when gated as either GFP^{+} or Gata3^{+} in the BAL (**A**) and mLN (**B**). Data is the combination of two individual experiments *P <0.05, **P <0.01.
