# Supplementary material for: Pulmonary Group 2 Innate Lymphoid Cell Phenotype Is Context Specific: Determining the Effect of Strain, Location, and Stimuli
Source: Front Immunol. 2020 Jan 22;10:3114. doi: 10.3389/fimmu.2019.03114 (PMC6987460; doi:10.3389/fimmu.2019.03114)
Supplement: Supplementary file 2 [file Table_1.pdf]

Table S1. Antibodies used to identify and phenotype mouse ILC2s.

Antigen	Clone	Fluorochrome	Manufacturer	Dilution	Stock concentration	Working concentration
CD11b	M1/70	APC	Biolegend	1:200	0.2mg/mL	1μg/mL
CD11c	N418	APC	Biolegend	1:100	0.2mg/mL	2μg/mL
CD127	A7R34	BV711	Biolegend	1:100	0.2mg/mL	2μg/mL
CD19	eBio 1D3	APC	Biolegend	1:100	0.2mg/mL	2μg/mL
CD25	PC61	BV510	Biolegend	1:50	0.5mg/mL	10ug/mL
CD3e	145-2C11	APC Cy7 (eFlour 780)	Biolegend	1:100	0.2mg/mL	2μg/mL
CD45	30-F11	AF700	Biolegend	1:100	0.5mg/mL	5ug/mL
CD5	53-7.3	APC	Biolegend	1:100	0.2mg/mL	2μg/mL
CD90.2	53-2.1	BV605	Biolegend	1:100	0.2mg/mL	2μg/mL
F4/80	BM8	APC	Biolegend	1:100	0.2mg/mL	2μg/mL
FCεR1	MAR1	APC	Biolegend	1:100	0.2mg/mL	2μg/mL
GATA3	TWAJ	PERCP e710	Thermo Fisher/ eBiosciences	1:25	60μg/mL	2.4μg/mL
GR-1	RB6-8C5	APC	Biolegend	1:100	0.2mg/mL	2μg/mL
KLRG1	2F1/KLRG1	PE Cy7	Biolegend	1:100	0.2mg/mL	2μg/mL
IL-13	eBio 13A	PE	Thermo Fisher/ eBiosciences	1:100	0.2mg/mL	2μg/mL

IL-5	TRFK5	BV421	Biolegend	1:100	0.2mg/mL	2µg/mL
Nkp46	29A1.4	PE/Dazzle™ 594	Biolegend	1:100	0.2mg/mL	2µg/mL
SCA-1	D7	BV785	Biolegend	1:100	0.2mg/mL	2µg/mL
ST2	DJ8	FITC	MD Biosciences	1:100	1mg/mL	10µg/mL
TCRβ	H57-597	APC	Biolegend	1:100	0.2mg/mL	2µg/mL
TCRγδ	eBio GL3	APC	Thermo Fisher/ eBiosciences	1:100	0.2mg/mL	2µg/mL
TER-119	TER-119	APC	Biolegend	1:100	0.2mg/mL	2µg/mL
ST2	DJ8	FITC	MD Biosciences	1:100	1mg/mL	100µg/mL
ST2	DJ8	PE	MD Biosciences	1:100	1mg/mL	100µg/mL
